# Macrocyclic NHC Ligands in Hoveyda-Type Ru Alkene Metathesis Catalysts: Only Sterics?

**DOI:** 10.1021/acs.inorgchem.5c03590

**Published:** 2025-09-15

**Authors:** Artur Brotons-Rufes, Sergio Posada-Pérez, Steven T. Diver, Albert Poater

**Affiliations:** a Institut de Química Computacional i Catàlisi, Departament de Química, 117394 Universitat de Girona, c/M^a^ Aurèlia Capmany 69, Girona, Catalonia 17003, Spain; b Department of General Chemistry: Algemene Chemie (ALGC), Vrije Universiteit Brussel, Pleinlaan 2, Brussel 1050, Belgium; c Department of Chemistry, University at Buffalo, the State University of New York, Amherst, New York 14260-3000, United States

## Abstract

The integration of macrocyclic structures within *N*-heterocyclic carbene (NHC) ligands in Hoveyda-type catalysts presents a pioneering strategy to enhance selectivity in cross-alkene metathesis. This approach falls within a promising paradigm for avoiding undesired homocouplings between reacting alkenes and introducing site-selectivity into the cross metathesis process. This paper presents a computational study that aims to provide an improved understanding of the impact of these ligand modifications on stability, sterics, and activity, focusing on the precatalyst activation and cross-metathesis. The traditional Hoveyda-type catalyst is compared alongside two recent macrocyclic systems for which experimental data is available. Higher activation barriers in the macrocyclic systems are consistent with the reduced activity observed in these systems. Additionally, a noncovalent interaction component was found to facilitate the selective pathway, in conjunction with the already expected steric descriptors. These results highlight the potential of macrocyclic NHC ligands to enhance catalyst performance, not only offering chemoselectivity based on alkene size, but a potential for stereoselectivity capacity arising from the macrocycle-induced electronic effects.

## Graphical abstract

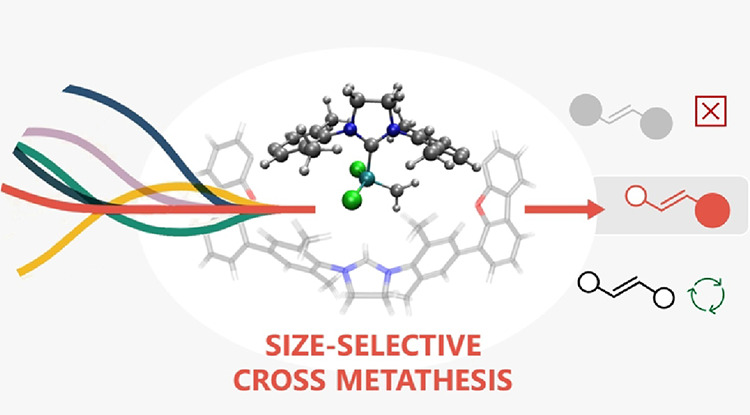

## Introduction

Metathesis reactions involve the scrambling of carbon–carbon (C–C) bonds, encompassing single, double, and triple C–C bonds for alkane,^1^ alkene,^2^ and alkyne metathesis,^3^ respectively. Particularly, alkene metathesis involves the reorganization of C–C double bonds in alkenes. Its origins date back to the early 1970s when Chauvin proposed a comprehensive mechanism elucidating the key steps involved.^4^ In this mechanism, the metallacycle was identified as the key intermediate, providing a foundation for further studies in this area. Subsequently, Grubbs and Schrock developed highly effective catalysts for this reaction,^5,6^ enabling its practical application.^7^ These catalysts not only enabled the practical application of this reaction but also opened up new avenues for enhancing the efficiency of the process,^8^ as well as preventing undesired deactivation reactions.

Cross-alkene metathesis stands out as a highly regarded coupling methodology by offering a direct and often cleaner approach to the formation of complex molecules.^9^ Unfortunately, the homocoupling of the terminal alkene substrates is a source of undesired product formation, which hinders the beneficial effects of its use. In this line, Grubbs and co-workers developed a valuable predictive model for cross metathesis, categorizing alkenes based on their reactivity.^10^ Among these groups, type 1 alkenes exhibit the highest reactivity and can undergo coupling with electron-deficient type 2 or type 3 alkenes.^11^ Highly reactive alkenes are expected to keep reacting in secondary metathesis events, affecting the chemoselectivity of the process. Thus, by choosing a proper combination of coupling partners, the cross-metathesis process can be expected to be mostly selective.

Given the broad applicability of type 1 alkenes as substrates, it is evident that synthetic chemists find themselves limited within this empirical model when using Grubbs’ type catalysts. This limitation is particularly true in the context of total synthesis scenarios involving advanced alkene intermediates requiring selective coupling or in cases where using an excess of one alkene is impractical.^12,13^

Addressing selectivity within functional groups poses a contemporary challenge in catalysis.^14,15^ In addition, the ability of a catalyst to selectively differentiate between two identical functional groups located in different parts of a molecule,^16^ referred to as site-selectivity, stands out as a crucial concern in modern catalysis. The introduction of an alkene metathesis catalyst capable of encapsulating substrates and discerning reactivity based on alkene size would represent a highly advantageous approach to controlling selectivity in cross-alkene metathesis.^17^ For alkene substrates varying in aggregate size, a size-selective catalyst has the potential to distinguish them (Scheme 1).

**Scheme 1:**
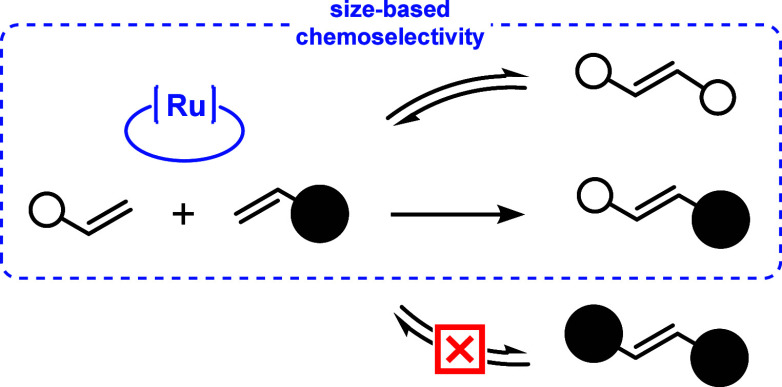
Selectivity in Cross-Alkene Metathesis Led by the Size

The discovery of a Ru carbene catalyst with this capability would significantly enhance the toolkit of task-specific Grubbs catalysts. The spatially restrictive interior of the macrocycle becomes pivotal since a selective metathesis reaction would become feasible if the macrocyclic Ru carbene catalysts can accommodate only the smaller alkene. Subsequently, the resulting alkylidene may externally react with the larger alkene, yielding the cross product. Notably, the small alkene can undergo reversible dimerization, a crucial aspect of the Grubbs selectivity model for alkene cross metathesis. Type 1 homodimers, even in their reversible state, even in their reversible state, remain reactive, perpetuating the production of active metal carbenes that lead to cross metathesis products.

In this line, Diver and co-workers opted to tackle the problem by introducing a macrocyclic N-heterocyclic carbene (NHC) in place of an acyclic NHC moiety such as H_2_IMes (Scheme 2).^18,19^ The literature about the combination of macrocycles and NHC ligands is scarce,^20^ and even more limited using ruthenium as a metal, where only the seminal works of Grela and co-workers,^21^ and Diver and co-workers,^18^ can be found. In 2020 it was reported the synthesis of a macrocyclic Ru carbene catalyst designed for selective cross-alkene metathesis (see Scheme 2, system **1**). This new catalyst exhibited distinct reactivity for different type 1 alkenes in homodimerization, a reactivity pattern correlated with the aggregate size of the allylic substituent. This modified reactivity profile enabled selective product formation in competition with cross-alkene metathesis between two different type 1 alkenes and *t*-butyl acrylate. In this previous report, the synthesis and reactivity of novel macrocyclic Grubbs catalysts was conducted, delving into homodimerization rates and exploring size selectivity through a competition cross-alkene metathesis with *t*-butyl acrylate. The observed selectivity in these reactions was attributed to the catalytic activity of the macrocyclic Ru carbene, capable of distinguishing alkenes based on their size. Overall, macrocyclic Ru carbenes were less reactive than the acyclic catalyst **3.**

**Scheme 2:**
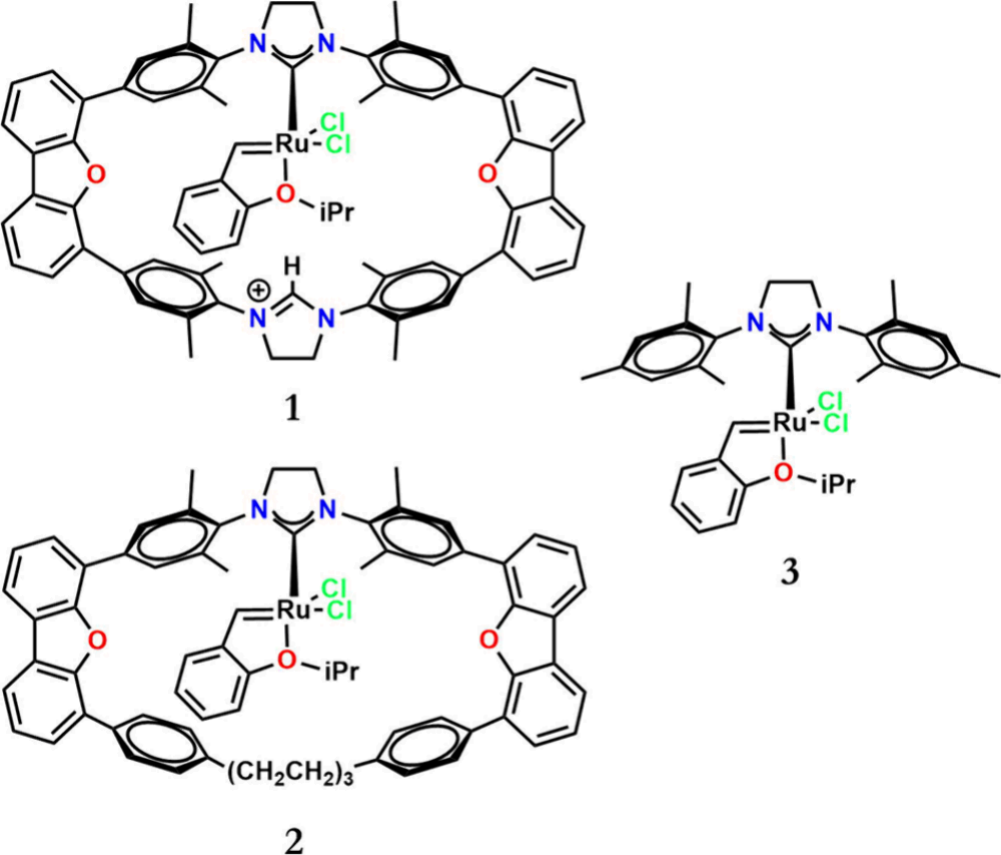
Systems of the Current Study: Cationic **1** and Neutral **2** Macrocyclic Hoveyda-Type Catalysts, and the Classical Hoveyda Catalyst **3**

Herein, by means of Density Functional Theory (DFT) calculations, we explain the reduced reactivity of these macrocyclic catalysts and find an unexpected interplay between stabilizing noncovalent interactions and steric destabilizations in critical reactive intermediates in the alkene metathesis catalytic cycle. We investigated catalysts **1** and **2** (Scheme 2) as compared to the classical Hoveyda catalyst **3**, with the primary goal of offering a detailed description of the effects introduced by the macrocyclic structure, their nature, and the relative stabilities of the stereoisomeric intermediates.^23^ To explain selectivity, we sought to better understand the interplay between the macrocyclic scaffold and the substituents of the alkenes. We examined the energies of all stereoisomeric metallacyclobutane intermediates, which also provides some insight into the potential stereoselectivity of the system, due to the introduced asymmetry in the coordination sphere.

## Computational Details

Starting geometries were taken from the XRD data in the 2020 experimental work.^19^ Conformational space exploration was carried out by means of the CREST program,^24^ from which the nine lowest energy conformations were selected for further optimization via DFT. All DFT calculations were carried out using the Gaussian09 package.^25^ Refined geometry optimizations employed the BP86 functional, i.e. the pure Generalized Gradient Approximation (GGA) functional of Becke and Perdew,^26^ with the inclusion of the Grimme D3 dispersion correction. These calculations were conducted using the all-electron double-ζ polarized def2-SVP basis set for light atoms,^27^ while for the accurate description of Ru atoms, the SDD basis set with effective-core potential for nonvalence electrons was used.^28^ All optimizations were performed without symmetry constraints, and the nature of the stationary points was verified through analytical frequency analysis. Gibbs energies at 298.15 K were computed using the electronic energy evaluated with the M06 functional,^29^ and the triple-ζ basis set def2-TZVP for all the atoms,^30^ except for Ru, which again was described employing the SDD basis set and pseudopotential combination. Additionally, solvent effects were estimated with the universal solvation model (SMD) by Cramer and Truhlar, using dichloromethane as the solvent.^31^ The reported Gibbs energies encompass electronic energies obtained at the M06/def2TZVP­(SMD­(DCM))∼SDD//BP86-D3/def2SVP ∼ SDD level of theory. These values were adjusted with zero-point vibrational energies, thermal corrections, and entropy effects computed at the BP86-D3/def2SVP ∼ SDD level, where the geometries were optimized. This protocol, or a very similar one, was adopted based on previous work in organometallic systems, particularly in olefin metathesis.^10,13,32^ Transition state geometries were localized via scans throughout the reaction coordinate connecting product and reactive. The imaginary frequency was then captured freezing the bonds involved in the rearrangement, and finally, the system was optimized fully relaxed. In some instances, the imaginary frequency could not be maintained in the last step, so the frozen mode structure was taken as an approximation. For the sake of consistency we checked if the Martin approach,^33^ of the overestimation of the entropy, could affect the results, however no significant differences were observed.

The steric analyses were carried out by means of the SambVca2.1 package of Cavallo and co-workers,^34^ using the definition of the buried volume, %V_Bur_. Since the first sphere around the metal is the region where the catalysis takes place, the standard radius of 3.5 Å was employed, enlarged up to 10 Å to take into account the nature of the macrocyclic ligands. On the other hand, to account for the noncovalent interactions, NCI plots of Contreras-Garcia et al. were computed.^35,36^ Those graphics have the capability of the NCI plots to check, verify and even evaluate qualitatively the strength of the noncovalent interactions (NCIs).^37^ 3D NCI plots show more attractive (in red) noncovalent interactions than repulsive ones (in blue) between pairs of H atoms close to space. The isocontour was obtained for a value of 0.5 on the reduced density gradient; whereas for the color scale we used the interval from −0.5 to 0.5 of the second density Hessian eigenvalue. 2D NCI plots allow the direct comparison of pairs of structures, unveiling the NCIs.

## Results and Discussion

Within the established mechanistic framework, the reaction pathway must begin with the precatalyst activation,^38^ which features a common benzylidene ligand bearing a chelating ortho-isopropoxy group (see Figure 1, top).^39^ The opening of the chelate is a critical step, typically associated with the highest energy barrier in the Gibbs energy profile and thus determining the overall catalytic activity. This process is known to be strongly influenced by the steric environment around the metal center.^40,41^

**Figure 1:**
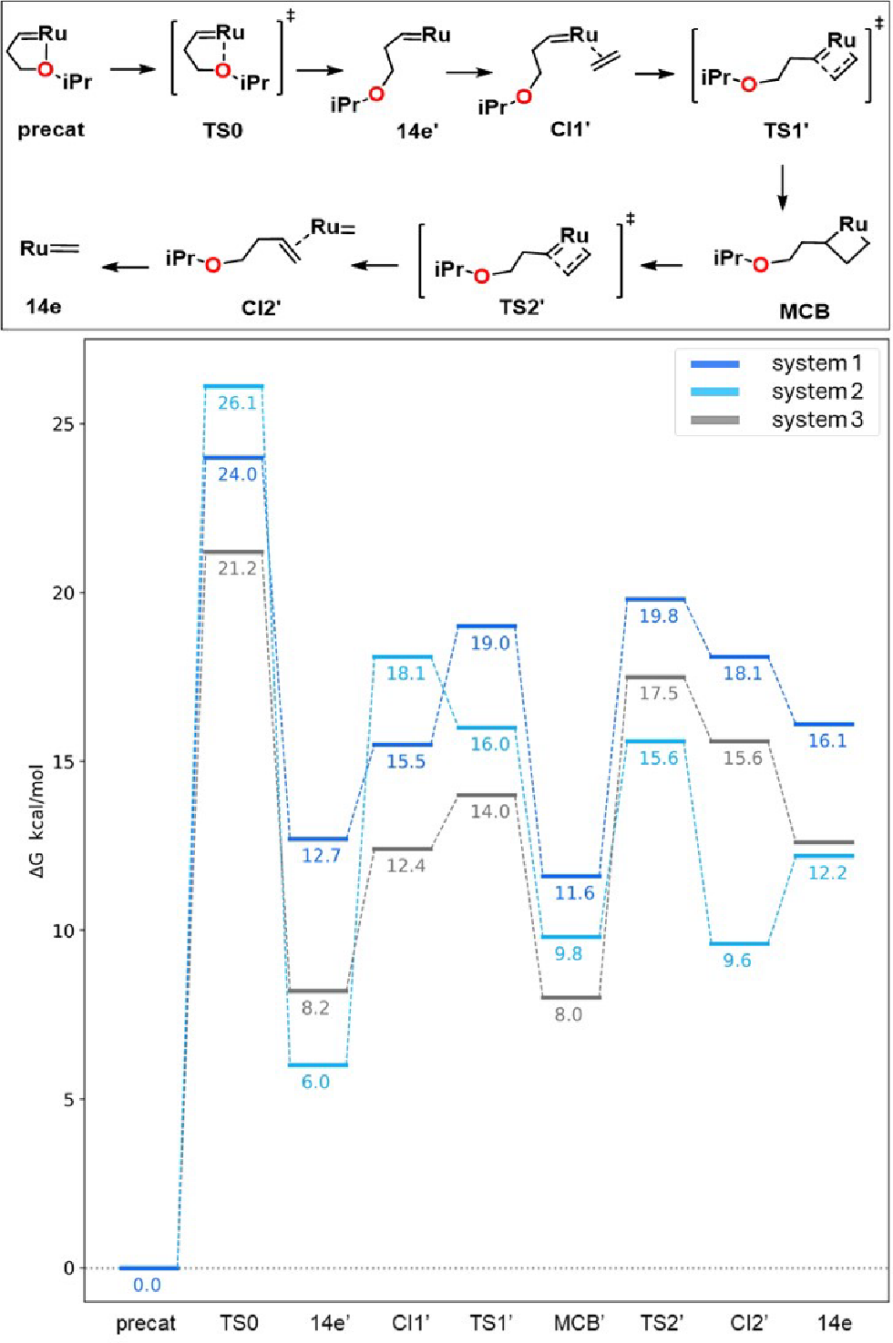
Activation mechanism for the cationic **1** and neutral **2** macrocyclic systems, and the Hoveyda catalyst **3** (for the neutral system there is a negative barrier once included the solvent effects). Gibbs energies at the M06/def2TZVP­(SMD­(DCM)∼SDD//BP86-D3/def2SVP ∼ SDD level of theory.

Hoveyda-type precatalyst activation is reported to potentially occur within three possible mechanistic pathways, referred to as the associative, dissociative and interchange.^38c,42^ Based on previous experimental and computational studies,^43^ NHC-derived catalysts are expected to have a predisposition for interchange and dissociative behaviors, probably both occurring in parallel.^44^ However, the relative dominance of one of them is significantly sensitive to the steric and electronic properties of the molecules involved. In general, the introduction of bulkier groups shows a decrease of the activation barriers (e.g., improved initiation rate by changing o-isopropoxy by o-phenoxy),^45^ favoring the dissociative process. Thus, only the dissociative pathway has been considered in our study, due to the more impelled center of the macrocyclic systems considered. Figure 1 (top) illustrates how the activation path was tackled.^46^ For the following OM-steps, an unsubstituted ethylene substrate was used, focusing on the role of the macrocycle for the macrocyclic cationic **1** and neutral **2** catalysts, compared with the classical Hoveyda catalyst **3**. Notice that the optimized TS0 was not found for the latter, and consequently, an approximated geometry from a linear transit analysis is used.

The energy profiles obtained for the three catalysts studied are shown in Figure 1 (below). The dissociative cleavage of the Ru–O bond, denoted as TS0, is found to be significantly more impeded for both the macrocyclic-containing systems **1** and **2** in comparison to the traditional system **3**. Surprisingly, the reactive carbene intermediate, denoted 14e’, is favored in the neutral macrocycle by 2.2 kcal/mol below the traditional system, despite the increased steric constraints. The fact that the cationic macrocycle is less stable suggests an increased flexibility of the macrocycle in system **2** over system **1**. This trend is mostly kept in the following steps, with the most stable system being at times the neutral system and at times the classic one, while the cationic system **1** tends to be the least stable during the process. Indeed, system **1** shows a slightly higher energy profile than the classic Hoveyda, but with a fairly similar shape, and similar kinetic barriers. The enhanced stability observed in the final intermediate of system **2**, with a margin of approximately 6 kcal/mol below system **3**, is a surprising result. Overall, when considering the effects of a macrocyclic moiety on the activation of the Hoveyda-type precatalyst, the diagram predicts a slower process in the modified catalyst, primarily attributed to the dihedral rotation mode required for chelate opening. This is in agreement with the experimental observations of Zhang and Diver,^19^ who found that the macrocyclic catalysts were much less reactive than the traditional system **3**. Specifically, knowing the relative rate constant (*k*_rel_) allows for the comparison between competitive reactions to compare the reactivity of different substrates, a *k*_rel_ 160 times greater was observed when comparing system **3** with system **1**. To further comprehend and rationalize these results, Figure 2 depicts the steric map analysis carried for the precatalysts **1**-**3** using SambVca2.1 of Cavallo and co-workers,^34^ where %V_Bur_ is the fraction of a sphere’s volume that is occupied by a given ligand when centered on a metal atom. It is calculated based on a standard sphere with a typical radius of 3.5 Å around the metal center that can be expanded when it is required.^47^ The total %V_Bur_ values unveil that the macrocyclic ligands impose more steric hindrance on the sphere around the metal center, but the %V_Bur_ values by quadrants are even more indicative, showing that two of them are nearly empty (i.e., values close to the quadrants of system **3**), and thus an alkene substrate can still access the metal center.^48^ The quadrants help visualize where bulky alkene substituents may be accommodated in the macrocyclic systems.

**Figure 2:**
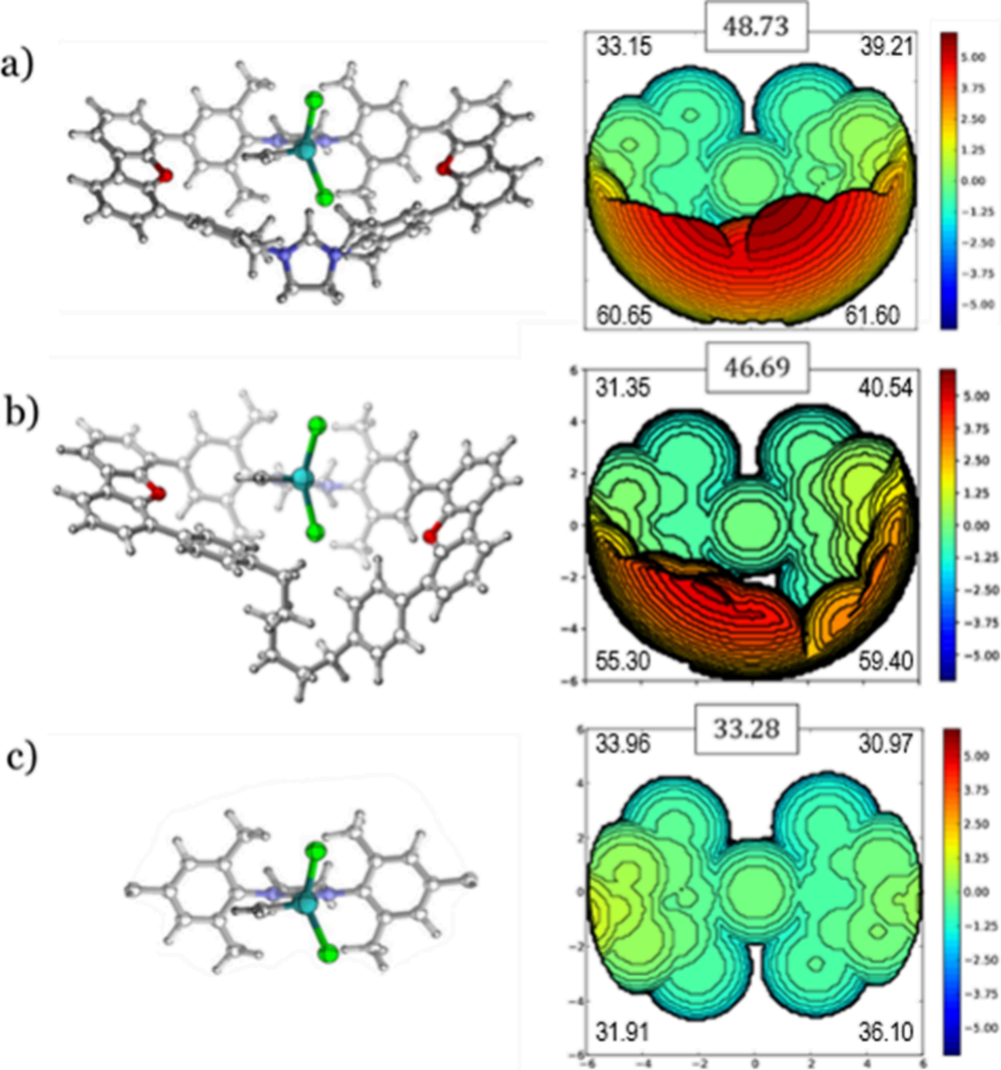
NHC ligands’ steric maps of the precatalysts (a) **1**; (b) **2**; and (c) **3**, with total %V_Bur_ and by quadrants on the xy plane (ruthenium as center, radius of 6.0 Å and Z axis defined by the N atoms of the NHC ligand; iso-contour curves given in Å). The *ortho*-isopropoxybenzylidene chelate is not added in the left 3D structures to ease the visualization.

Interestingly, neutral system **2** does not present the same steric hindrance in left and right quadrants,^49^ instead displaying a distinctive unsymmetrical buried volume profile. The origin of it is a folding of the six-membered methylene chain, see Figure 2c and Figure 3, which was also found in the experimental XRD of the original articles.^18^ Additionally, in contrast to catalyst **1**, the chain was found to be very flexible, also observed in the meta-dynamics obtained by CREST. This has a significant impact on the relative %V_Bur_ of system **2**, depending on the position of the carbene moiety, as illustrated in Figure 3. It is evident that, while a modest energy disparity is observed between these two isomers, more substantial variations are anticipated in the presence of bulky substituents, thereby elucidating the more fluctuating energies depicted in Figure 1. Consequently, it can be inferred that the %V_Bur_ index alone is not a suitable descriptor for system **2**, unless appropriate preoptimization and conformational search protocols are employed.^50^

**Figure 3:**
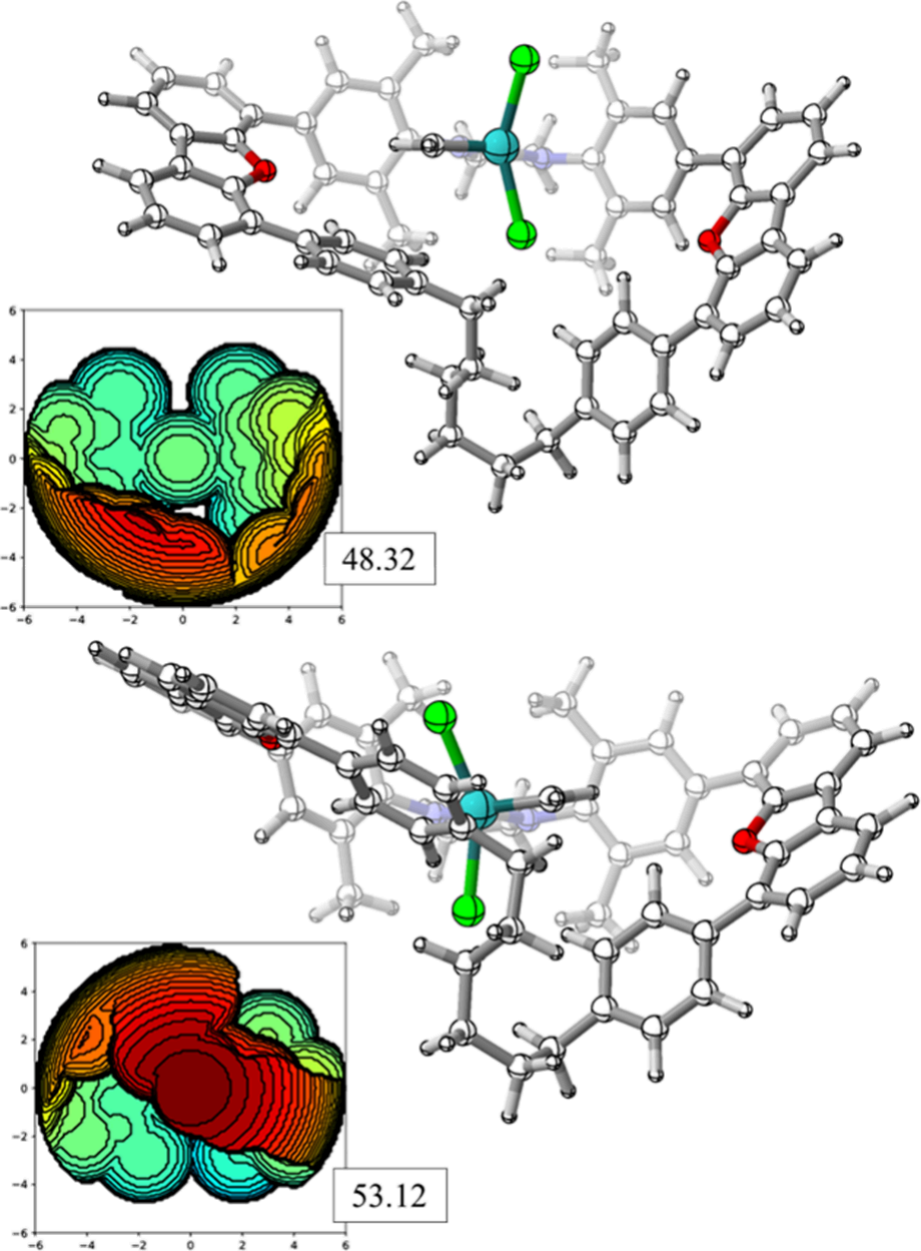
Steric maps of the 14e methylidene intermediate for complex **2**; left-sided (top) and right-sided (bottom), with the corresponding steric maps and %V_Bur_ values.

In the framework of cross-metathesis, our investigation continued with the introduction of substituted olefins into the model. In this phase of the study, only cationic system **1** was considered since it is the only one with experimental data available. Following the cross metathesis reported by Diver,^19^ a *t*-butyl ester olefin was reacted with two other alkenes that varied considerably in the steric demand of their substituents: a bulky 4,4,4-triphenyl-1-butene and 1-hexene. However, due to the steric pressure in one-half of the coordination sphere, the different possible conformations had to be considered, including the relative orientation of the substituents toward the macrocycle. Scheme 3 shows a schematic representation of the different stereoisomers of the alkylidene complexes. Prior to the calculations, the OUT-*cis* configuration was identified as the one more convenient, since both alkenes are orientated in the opposite side of the macrocycle.^51^ However, we found a potentially favorable interaction between the ester group and the C2 hydrogen of the electron-deficient azolium moiety in the IN-type conformations.

**Scheme 3:**
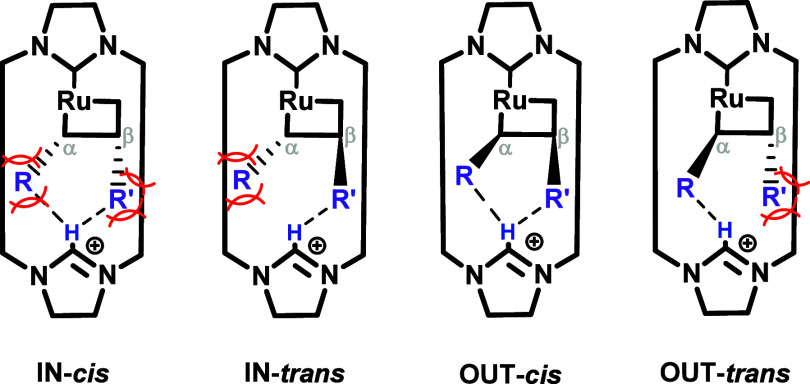
. Stereoisomer Conformation of Ruthenacyclobutanes Studied in the Competitive Studies (**R** & **R’** Either (CH_2_)_3_CH_3_ (Alkyl) or CH_2_CPh_3_ (Aryl))^a^

a Red lines are used to represent the steric hindrance observed in the corresponding stereoisomers.

First, the ester-ylidene intermediate was optimized. In the most stable structure found, the ylidene moiety’s hydrogen is in the same plane as the Ru-NHC bond (see Scheme 4, left). Such dihedral angle orientation was found to be enforced by the steric constraints of the *t*-butyl substituent. In addition, the ester’s carboxyl bond was oriented to the azolium hydrogen with a distance of 1.95 Å, which fits within the range of H-bonds. Indeed, when enforcing different dihedral positions where the H-bond would not be present, only one stereoisomer could be optimized (see Scheme 4, right). In it, the *t*-butyl group is found clashing with the macrocycle, increasing the energy of the system increased by more than 15 kcal/mol. This energy difference between isomers is attributed to the simultaneous increased steric pressure and the loss of the internal H-bond.

**Scheme 4:**
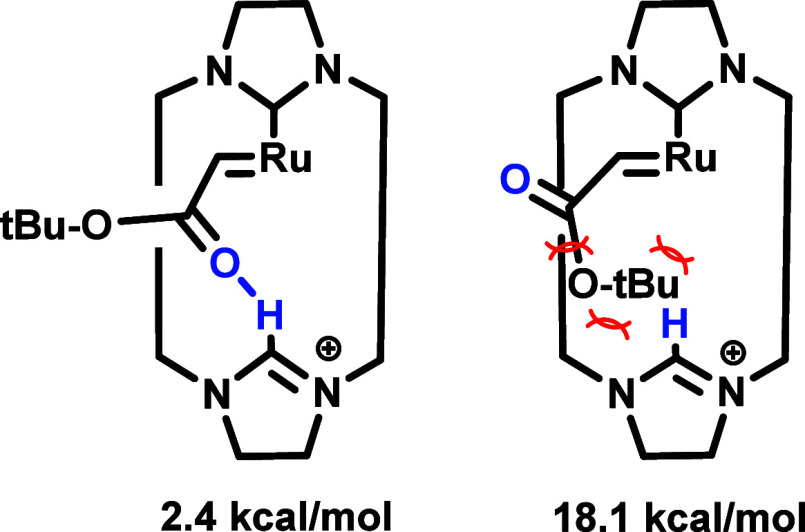
Representation of the *t*-Butyl Ester Ylidene Conformers Studied: the Ester Group Oriented Outward from the Cycle (Left) and Inward (Right), with Gibbs Energies Reported Relative to **precat** **-1** ^a^

a Red lines indicate steric hindrance

Next, the ruthenacyclobutane intermediates were optimized. Starting from the latter ester-ylidene, four stereoisomers were considered for both coupling alkenes; ‘alkyl’ is the alkene with lower steric demand, whereas ‘aryl’ refers to the largest alkene (see Scheme 3). In Table 1, the R group (α) is CO_2_tBu and entries 1 and 2 compare the incoming alkene as R’ (β), either −CPh_3_ or n-C_4_H_9_. The alternative pathway involving the initial reaction of 1-hexene with the catalyst followed by cross metathesis with *t*-butyl acrylate was also considered, thus placing CO_2_tBu in the β-position of the metallacyclobutane and C_4_H_9_ in the α position, as shown in entry 3. However, the corresponding triphenyl complexes did not optimize, since the aromatic rings clash with the second heterocyclic carbene moiety when in the α-position, so energetic comparisons between alkenes of different sizes could not be made. Similarly, *cis*-dichloride alternatives were considered, i.e. the metallacycle’s plane *cis* to the Ru-NHC bond. However, the calculations only converged for an alkyl containing geometry and, despite being more stable than its original IN-*cis* bottom bound counterpart (*i.e. trans*-dichloride),^52^ the proximity of the substituents made it impossible to picture any viable transition state following (or preceding) it. Consequently, these *cis*-dichloride isomers were completely excluded from the subsequent analysis.

**Table 1:** Relative Gibbs Energies (in kcal/mol) Calculated at the M06/def2TZVP­(SMD­(DCM))∼SDD//BP86-D3/def2SVP ∼ SDD Level of Theory, Obtained for the Ruthenacyclobutanes Stereoisomers for the Alkyl and Aryl Olefins

	IN-*cis*	IN-*trans*	OUT-*cis*	OUT-*trans*
**-CPh**_**3**_ **(R’)**	19.5	4.1	14.6	16.9
**-(CH**_**2**_ **)**_**5**_ **CH**_**3**_ **(R’)**	16.3	–2.4	9.4	2.8
**-(CH**_**2**_ **)**_**5**_ **CH**_**3**_ **(R)**	0.9	2.9	2.4	2.5
**%V**_**Bur**_^**–CPh3(R’)**^	34.26	29.09	26.45	28.69
**%V**_**Bur**_^**–(CH2)5CH3(R’)**^	29.31	25.84	25.67	24.62
**%V**_**Bur**_^**–(CH2)5CH3(R)**^	22.93	26.00	22.82	22.89

Overall, the lowest energy set corresponds to the latter alpha-alkyl set, with energies below 3 kcal/mol. Although in terms of reactivity the lowest energy path is the only one that is meaningful, comparison of the isomer energies was done in order to understand in detail the role of the macrocycle. In this line, very different behaviors were found within each regioisomer set. For instance, while the most stable intermediates of the alpha alkyl (*n-*C_4_H_9_ (R)) configuration corresponds to the IN-*cis* stereoisomer, the homologous IN-*cis* were the least stable ones in the other conformation sets (16.3 and 19.5 kcal/mol for the *n*-C_4_H_9_ (R’) and −CPh_3_ (R’), respectively). Conversely, the least stable IN-*trans* configuration in the *n-C*_4_ *H*_9_ (R) set became the most stable option for the other two sets. Indeed, the IN-*trans*-beta-alkyl was the most stable metallacyclobutane of all (i.e., −2.4 kcal/mol). This could be explained by the previously mentioned hydrogen bond. In this line, when looking at the %V_Bur_ carried for all the metallacycle intermediates (Table 1), although there is a clear increase in the energy when occupied volume increases, the descriptor seems to fail within close values. Indeed, the R^2^ value correlation between the nine-metallacyclobutane and the %V_Bur_ significantly improves when removing only the two IN-*trans* α-ester intermediates from the regression, going from 0.559 to 0.883 (see Table S11 in the SI for the corresponding plots). Another mild source of destabilization that was not properly captured by the %V_Bur_ was the steric conflict between the alkene substituents and the exterior part of the macrocycle. This is especially true in the alpha positioning of the bulkier *t*-butyl group of the ester, which puts it in close proximity to the ‘walls’ of the macrocycle.

As the relative energies correlation using solely steric descriptors proved unsatisfactory, reduced density gradient analysis of the ruthenacyclobutanes was conducted by means of the NCIPlots software to better assess potential noncovalent contributions. The results obtained from this analysis indicate the presence of several midstabilizing interactions (at the range of van der Waals and π-π stacking) between the alkene substituents (in the geometries with the substituents in *cis*), and between these and the macrocyclic ligand (see Figure 4 (left) for two examples and SI for the rest). The 3D surfaces reveal the existence of interactions from the aromatic rings of the triphenylmethyl and the dibenzofuran moieties, as well as from the phenyl-type rings and the methyl of the *t*-butyl ester. Additionally, a hydrogen bond is formed between the ester’s carbonyl group and the cationic NHC. The 2D-NCI plots (see Figure 4, right) reveal the nature of these interactions as peaks around −0.03 (see the example with a H-bond corresponding to IN-*cis* alpha-alky (top) and another one without a H-bond OUT-*cis* (bottom)). This was confirmed for all other geometries and is documented in the SI. It is notable that there are other minor bands, but the one attributed to the H-bond is the one of higher magnitude. However, the sum of these minor contributions, which come mostly from the aromatic rings of the macrocyclic moiety with the substituents, may affect the relative stability of each intermediate. In this sense, a comparison between the 2D plots should help to quantify these accumulated effects. Ideally, this would be achieved by integrating the areas, but in this case, a more semiquantitative approach has been adopted for the sake of simplicity.

**Figure 4:**
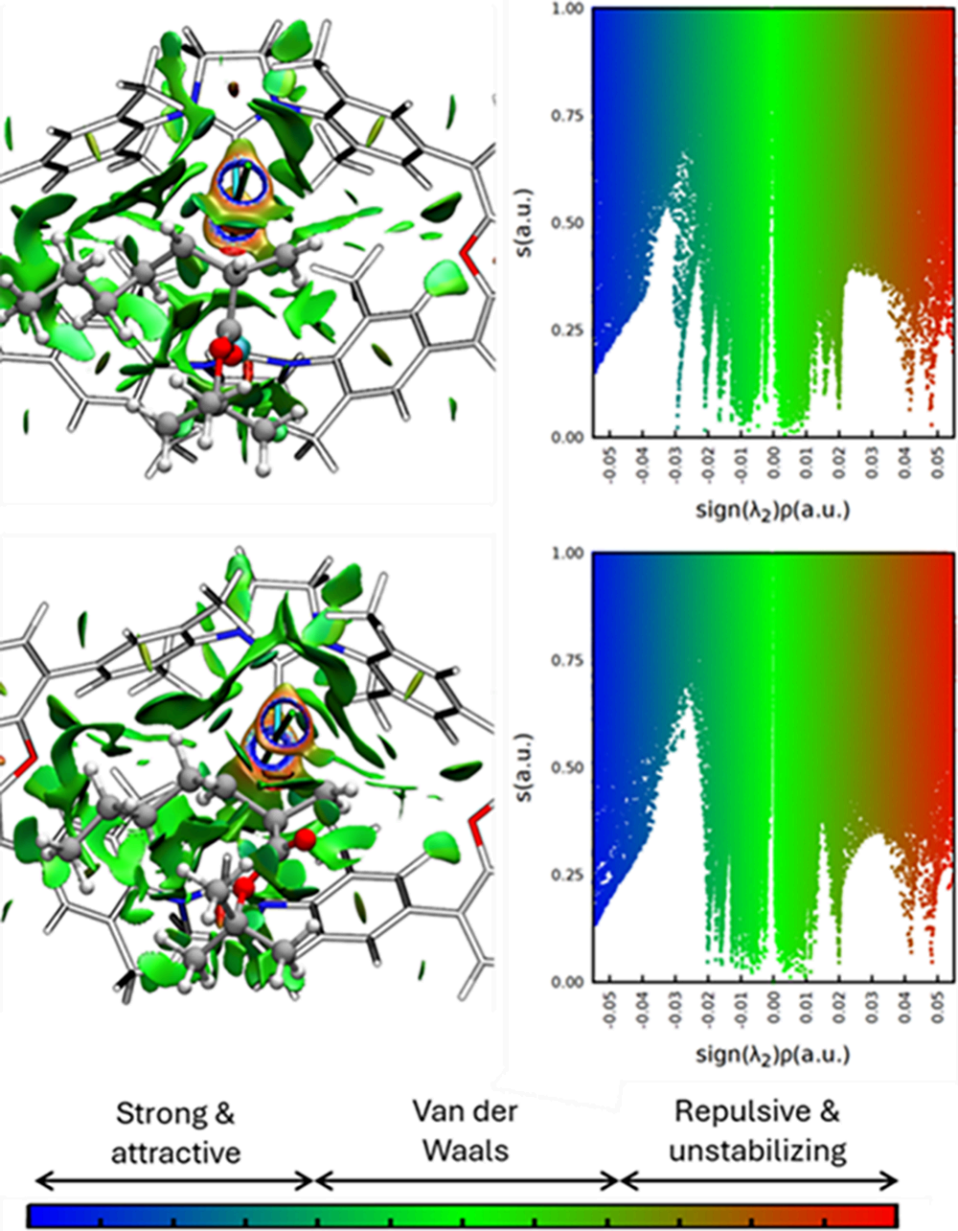
Tridimensional and bidimensional NCI plots of the IN-*cis* (supra) and OUT-*cis* (infra) alkyl metallacyclobutanes (for the 3D plots: the isocontour is obtained for a value of 0.5 on the reduced density gradient, and, for the color scale, we used the interval from – 0.5 to 0.5 of the second density Hessian eigenvalue, going from red (repulsive) to blue (attractive); for the 2D plots: reduced density gradient (s) vs sign (λ_2_)­ρ, in a.u.).

Superposition of the bidimensional plots helps to qualitatively identify changes in intramolecular interactions between the former alkenes and their influence on the subsequent macrocycle for the different isomers.^53^ For instance, when comparing two cases with similar %V_Bur_ but different stability, Figure 5 (top), a significant increase in the left side of the bidimensional NCI is observed.^54^ These (−)-sign interactions correspond to stabilizing interactions, including the H-bond (appearing as a sharp signal around −0.03), which allows us to justify the increased stability. Other comparisons of the alpha-alkyl substituents that further support the impact of NCI on the energies are shown in Figure 5 (middle: IN-*trans vs* OUT-*cis* & infra: IN-*cis vs* IN-*trans*). The former pair shows two almost identical bidimensional plots, while significant differences in the steric congestion (Figure 5 top). Thus, upon equivalent NCI scenarios, the relative stability is successfully described only with the %V_Bur_. This case is complementary to the one of Figure 5 (top), where only the NCI picture was changing significantly. The picture is completed when the two different effects change, which is the case of IN-*cis* vs IN-*trans* of the alpha-alkyl set. These two stereoisomers, which are the ones with the highest energy disparity, are also the ones showing the accumulative contributions of the sterics and intramolecular interactions.^55,56^ The IN-*cis*, the most stable one, has a relatively lower %V_Bur_ at the same time that shows a significant increase in the (−)-sign side of the NCI interactions. The contrary is observed for the IN-*trans* homologue, becoming the least stable of the conformation set.

**Figure 5:**
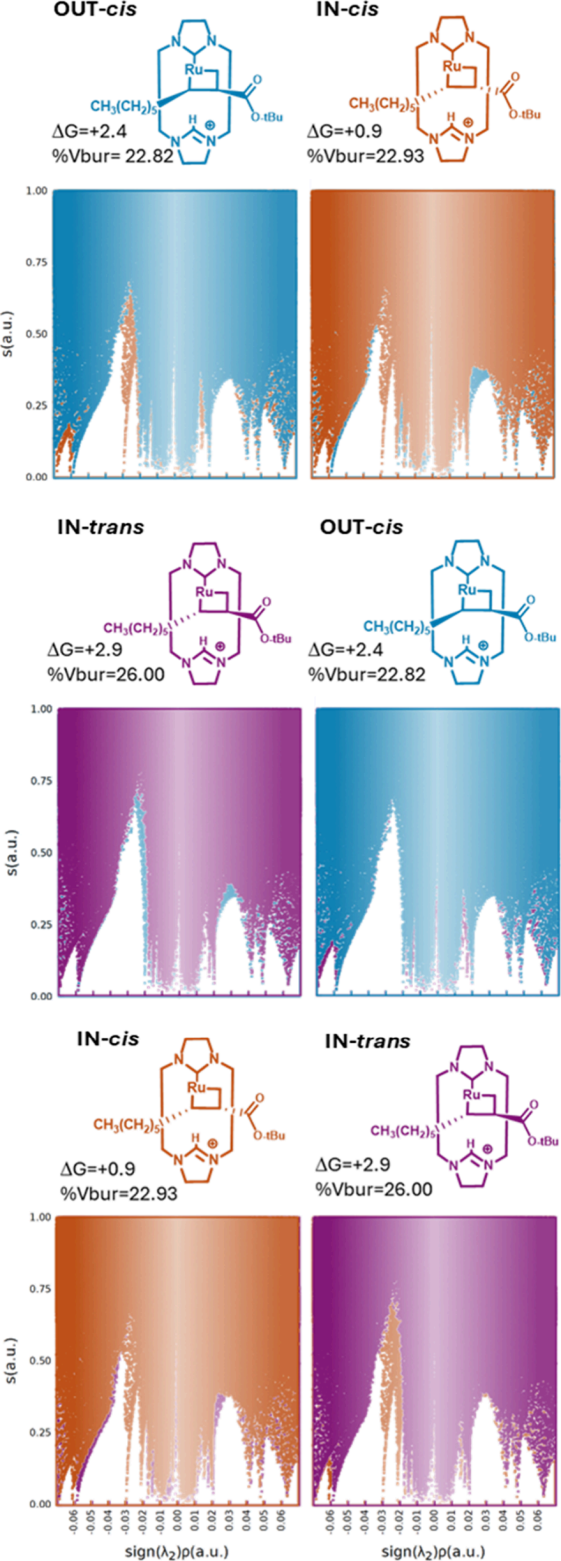
Superposition of the bidimensional NCI plots of the stereoisomers represented above, together with the %V_Bur_ values and the relative stability in kcal/mol.

Looking at the systems bearing the larger −CPh_3_ group, their relative energies can be justified in an equivalent way. For the similar %V_Bur_ configurations, OUT-*cis* (26.45%) and OUT-*trans* (28.69%), the stability follows the steric hindrance within the macrocycle (12.9 and 12.2 kcal/mol respectively), except for the IN-*trans* case (29.09%), due to the presence of a hydrogen bond (Figure 6). However, the intramolecular stabilizing interactions cannot compensate for the excessive increase in steric congestion in IN-*cis*, where the two bulky substituents face inward of the macrocyclic ligand. As a result, this isomer is the least stable among the series.

**Figure 6:**
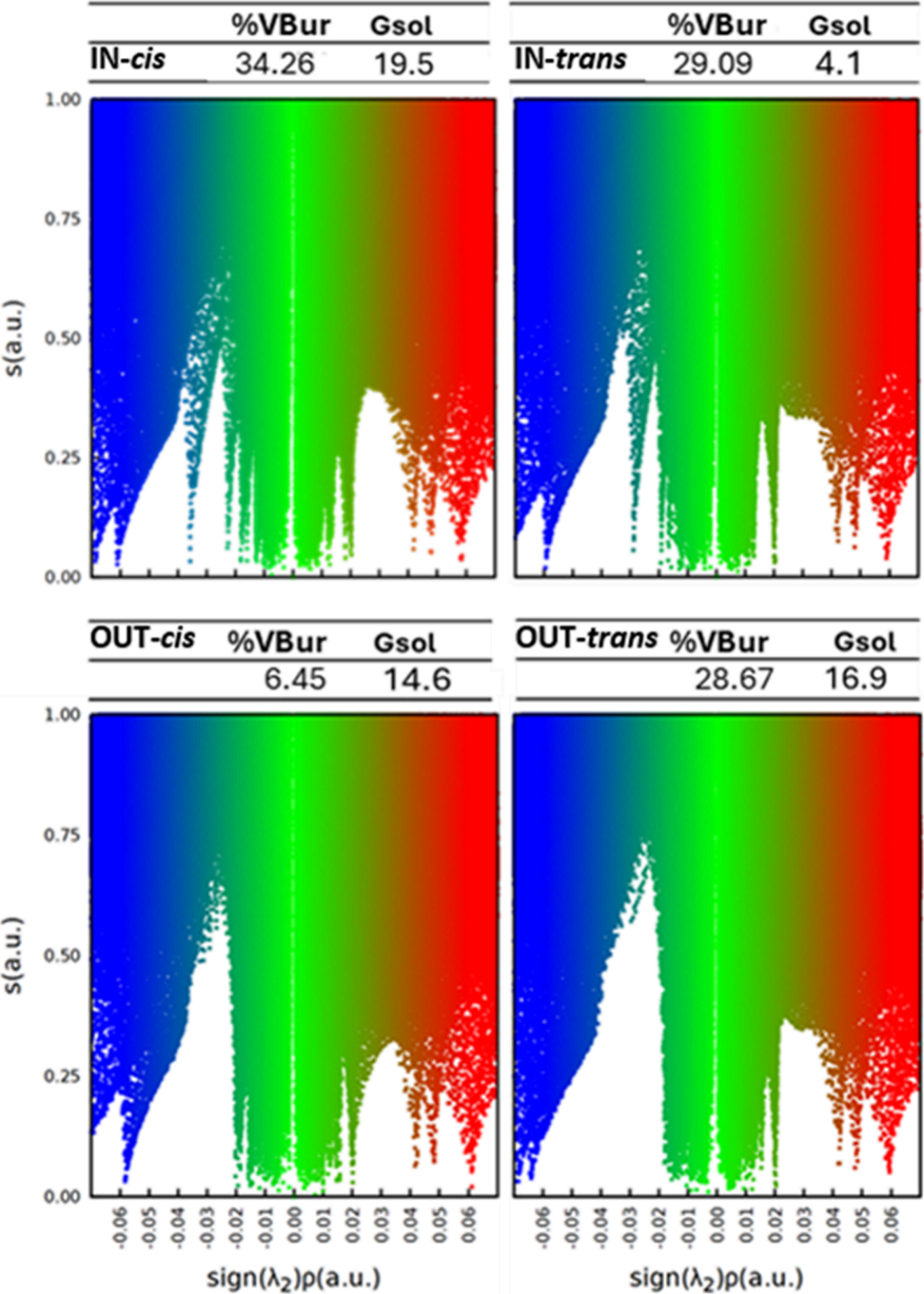
Bidimensional NCI plots of the metallacycle stereoisomers containing the aryl-alkene (**-CPh**_**3**_ **(R’)**), together with the %V_Bur_ values and the relative stability in kcal/mol. From these last observations, it can be inferred that the intramolecular noncovalent interactions can compensate the steric repulsions to a certain extent, thus showing the different weight of each effect on the final energy of the molecule.

To sum up, the NCI analysis helped rationalize the higher stability of certain MCB intermediates, despite their high %V_Bur_ values. Although MCB energies do not directly determine the kinetics, we observe a clear correlation between the stability of these intermediates and the energies of their corresponding transition states (see Figure 7), with two notable exceptions: IN-*trans* β-triphenylmethyl and IN-*trans* α-alkyl.

**Figure 7:**
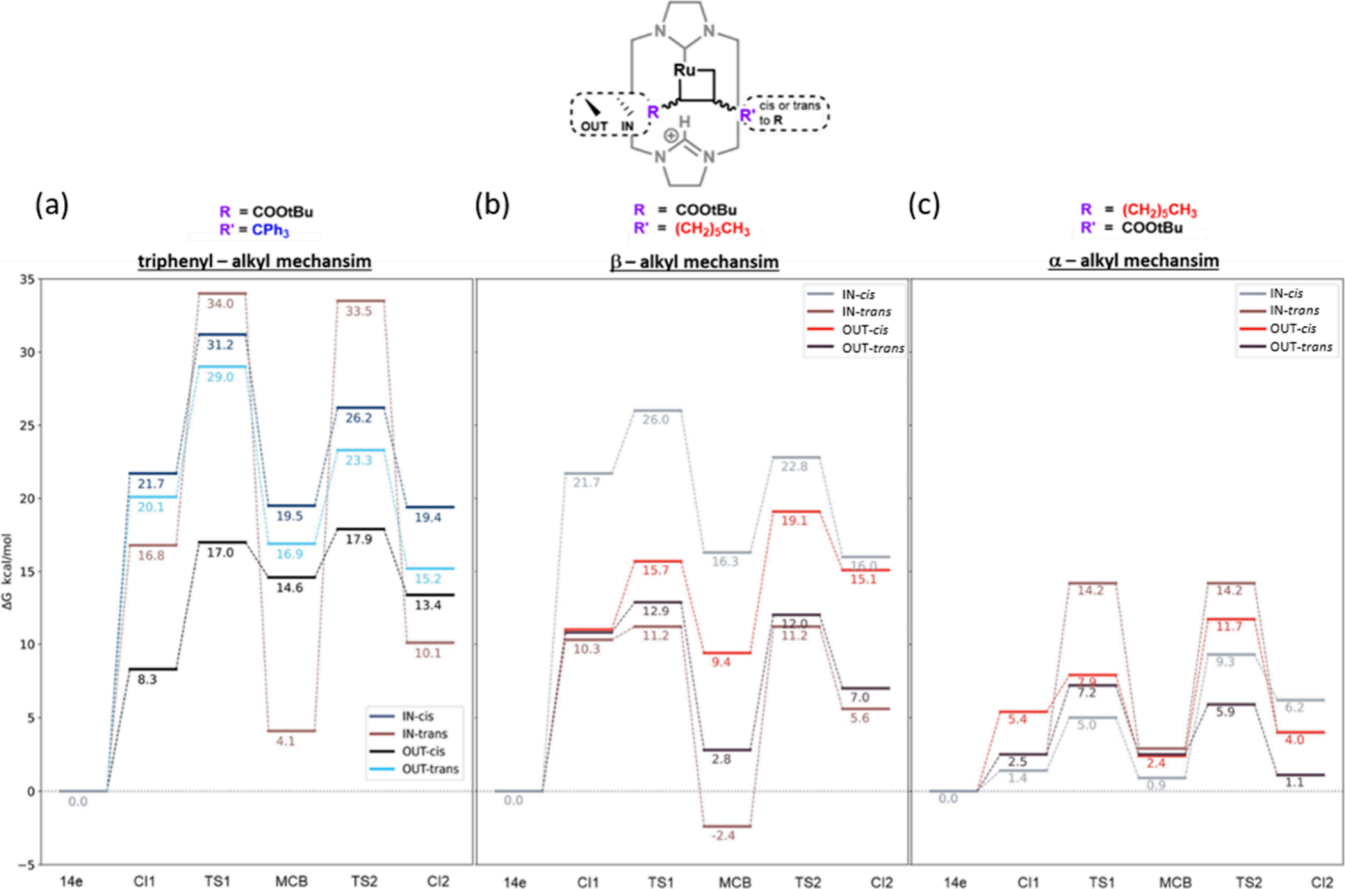
Energy diagrams (relative Gibbs energies in kcal/mol calculated at the M06/def2TZVP­(SMD­(DCM))∼SDD//BP86-D3/def2SVP ∼ SDD level of theory) of the cross-metathesis reaction of the *t*-butyl ester with (a) the triphenyl-alkene, (b) the alkyl-group and (c) its regioisomer, alpha-alkyl (approximated transition states from linear transit analyses are indicated by an asterisk (*)).

Finally, the overall cross-metathesis mechanism is considered (Scheme 5), with the corresponding energies presented in Figure 7. It should be noted that some transition states could not be optimized and are marked with an asterisk in the energy diagram. In general, the relative energies of the barriers corresponding to the formation (**TS1**) and cycloreversion (**TS2**) of the ruthenacyclobutane intermediates follow, in most cases, the same trend as observed for the metallacycles. However, significant deviations are observed when comparing stereoisomers within the same system.

**Scheme 5:**
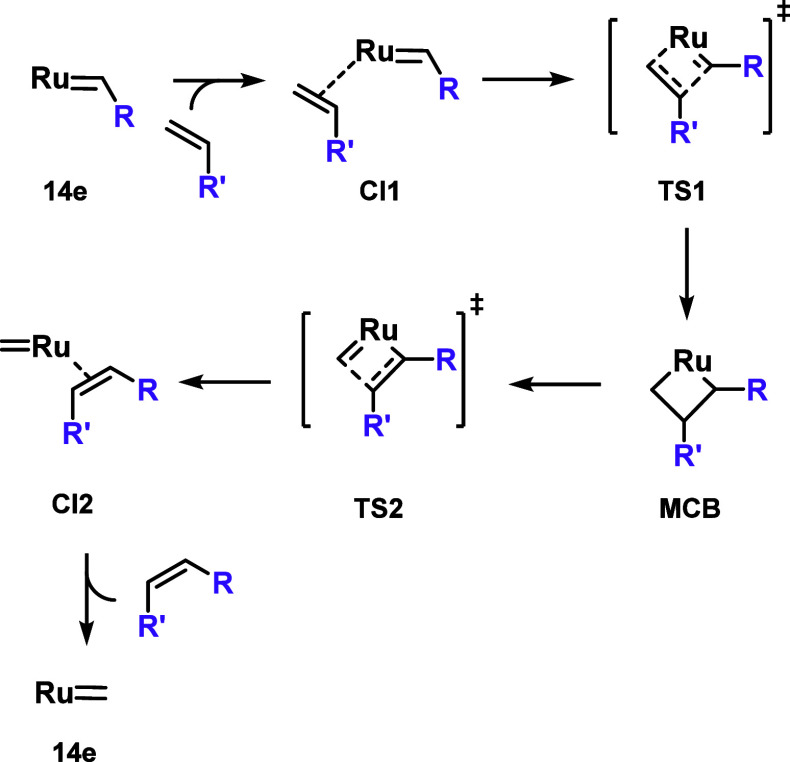
Scheme of the Olefin Metathesis Reaction Path Used in the Calculations with Corresponding Energies in Figure 7 ^a^

a (**R** & **R’** either (CH_2_)_3_CH_3_ (alkyl) or CH_2_CPh_3_ (aryl))

If the methyl acrylate reacts first, selectivity based on alkene size can be understood by comparing energies in Figure 7a and 7b. The lowest energy pathway of the β-triphenyl substituted system via the OUT-cis MCB has a higher energy pathway than that for the β-C4H9 substituent, which favors the IN-trans MCB.

Overall, the α-alkyl regioisomer set exhibits a much lower energy profile making this the most likely catalytic reaction pathway. Differences emerge as early as the coordination intermediate **CI1**, which already shows a lower energy compared to the other two isomeric pathways. This early stabilization lowers the energy landscape for the subsequent steps, contributing to more favorable kinetics. Both observations can be rationalized by considering the elliptical shape of the cavity in system **1** (see Scheme 2 or Figure 2). This geometry makes the α-position more sterically constrained than the β-position, favoring rearrangements where the bulkier group occupies the β-position. For Scheme 7c, this corresponds to the ester group. In contrast, for the bulkier aryl system, the triphenyl group is sterically more demanding, rendering the α-aryl rearrangement unfeasible due to clashes with the ligand periphery. The exact energies of this pathway could not be compared to that of the α-alkyl mechanism in Scheme 7c since the geometries could not be optimized.

Overall, the much higher energy of the aryl pathway is consistent with experimental observation,^19^ in which no triphenyl product was detected. The calculation of the different substituent orientations was necessary to identify the lowest energy mechanism, specifically the alpha-alkyl path, which is the most relevant in terms of reactivity. However, as previously stated, the other computed geometries were also employed to gain a more complete understanding of system **1**’s behavior. In this line, although mostly higher in energy, alkyl and aryl paths show some unexpectedly stable intermediates. As described before for the MCB, some of these cases can be explained by a sporadic match of the substituents with the macrocycle’s shape, which simultaneously reduces the steric hindrance while introducing stabilizing interactions. However, this seems to be restricted to these specific metallacyclobutane intermediates. For instance, a huge gap is found between the transition energies and the MCB at the aryl-IN-*trans* path (see Figure 7, left, brown line). Footnote: It should be noted that both of the TSs in this conformation are approximated, and thus, they cannot be completely trusted. The superior stability of the MCB intermediate can be attributed to both a favorable placement of the bulky triphenyl group outside of the cavity and allowing the ester group to form the hydrogen bond with the ligand. However, this unique situation seems to be completely lost when moving toward cycloreversion transition states due to the greater distances between groups in these latter intermediates. Indeed, this may indicate that the IN-*trans* aryl is not experimentally accessible. A similar situation is found in the IN-*trans* alkyl path (see Figure 7, middle, brown line), where the MCB becomes the most stable isomer of all, with the respective TS energies decreasing correspondingly.

## Conclusions

To sum up, the study of two macrocyclic Ru-NHC based catalysts, cationic and neutral, initiated with the precatalyst activation to be compared with the traditional Hoveyda catalyst, employing a common benzylidene with an ortho-isopropoxy group. The chelate opening, crucial for catalyst activity, is influenced by steric factors around the metal center. The activation pathways for Hoveyda-type precatalysts were explored, focusing on the dissociative mechanism. The incorporation of the macrocycle structure resulted in a significant increase in the activation barrier, which may explain the reported decrease of the catalytic activity. Steric hindrance analysis revealed differences between cationic and neutral systems. Moreover, the neutral system displayed more flexibility, requiring appropriate exploration of the conformational space.

Selective cross-alkene metathesis with substituted olefins was studied in the macrocyclic systems. The cationic system exhibited preferential chemoselectivity for smaller alkyl-substituted olefins over larger aryl-substituted olefins. Several different metallacyclobutane conformations were considered. Due to different stabilities of metallacyclobutane intermediates that did not correlate with sterics based on buried volume, noncovalent interaction analyses showed that some intermediates had both stabilizing non covalent interactions and steric strain. Indeed, this analysis revealed a secondary mechanism acting within the macrocycle, consisting of noncovalent interactions between the alkenes and the ligand scaffold.

Finally, the energies of the overall catalytic reaction were studied for the selective cross-alkene metathesis between alkenes and *t-*butyl acrylate. The presence of the *t*-butyl ester in the α-position was compared with both the smaller and larger alkene, and the energy of the reaction with the smaller alkene was lower. In addition, the smaller alkene likely reacts with the macrocyclic catalysts first, then undergoes a lower energy reaction to accommodate the *t*-butyl ester in the β-position of the metallacyclobutane. Since the energy of the pathway for the bulkier alkene could not be determined, a direct comparison could not be made for this more likely selective alkene metathesis pathway. In detail, this study reveals how macrocyclic Ru–NHC catalysts control olefin metathesis selectivity through a combination of steric and noncovalent effects. DFT calculations show that the α-alkyl pathway is the lowest-energy route, consistent with the absence of triphenyl products experimentally. Metallacyclobutane intermediates exhibit energy differences up to ∼ 20 kcal/mol, governed by buried volume and stabilizing H-bonds. Reduced density gradient (NCI) analysis highlights weak interactions that modulate intermediate stability beyond steric descriptors alone. These insights clarify the origin of catalyst selectivity and offer quantitative guidance, such as %V_Bur_ thresholds and identifiable intramolecular contacts,^57^ for future Ru–NHC catalyst design.

These studies shed light on the interplay of the sterically destabilizing interactions imposed by the bulky macrocycle, coupled with the unexpected stabilization possible in metallacyclobutane intermediates. These interactions were revealed through careful analysis of the buried volume and the noncovalent interactions. A better understanding of these interactions may potentially be exploited for alternative selectivity strategies and future catalyst design.

## Supplementary Material





## Notes

Absolute energies and additional details for steric maps and NCI plots; conformational seach and correlations (PDF)
XYZ coordinates (XYZ)
