# Supplementary material for: Macrocyclic NHC Ligands in Hoveyda-Type Ru Alkene Metathesis Catalysts: Only Sterics?
Source: Inorg Chem. 2025 Sep 15;64(38):19485–96. doi: 10.1021/acs.inorgchem.5c03590 (PMC12486211; doi:10.1021/acs.inorgchem.5c03590)

Macrocyclic NHC Ligands in Hoveyda Type Ru Alkene Metathesis Catalysts: Only Sterics?

Artur Brotons-Rufes,[a] Sergio Posada-Pérez,[a,b]* Steven T. Diver,[c]* and Albert Poater[a]*

[a] Albert Poater, Institut de Química Computacional i Catàlisi, Departament de Química, Universitat de Girona, c/Mª Aurèlia Capmany 69, 17003 Girona, Catalonia, Spain. E-mail: albert.poater@udg.edu

[b] Department of General Chemistry: Algemene Chemie (ALGC), Vrije Universiteit Brussel, Pleinlaan 2, 1050 Brussel, Belgium. Email: sergio.posada.perez@vub.be

[c] Steven T. Diver, Department of Chemistry, University at Buffalo, the State University of New York, Amherst, New York 14260-3000, United States. Email: diver@buffalo.edu

Additional information/details

Summary

Energy Calculation Tables S4

Table S1. Conformational search and preoptimization for system **1** (via CREST + GAUSSIAN09), absolute energies in a.u. Initial geometry obtained from optimization of the XRD precatalyst structure upon removing the chelate chain of the ylidene. S4

Table S2. Conformational search and preoptimization for system **2** (via CREST + GAUSSIAN09), absolute energies in a.u. Initial geometry obtained from optimization of the XRD precatalyst structure upon removing the chelate chain of the ylidene. S4

Table S3. Precatalyst path optimization for system **1** (GAUSSIAN09), absolute energies in a.u...S5

Table S4. Precatalyst path optimization for system **2** (GAUSSIAN09), absolute energies in a.u...S5

Table S5. Precatalyst path optimization for system **3** (GAUSSIAN09), absolute energies in a.u...S6

Table S6. Summary of the relative energies for the IN and OUT conformations (GAUSSIAN09), in kcal/mol. S6

Steric maps S7

Table S7. Steric maps for COOtBu-alkyl (radius of 10 Å). The isocontour curves of the steric maps are in Å. The xz plane is the mean plane of the NHC ring, whereas the yz plane is the plane orthogonal to the mean plane of the NHC ring, and passing through the carbene C atom of the NHC ring. The metal is at the origin. S7

Table S8. Steric maps for COOtBu-cph3 (radius of 10 Å). The isocontour curves of the steric maps are in Å. The xz plane is the mean plane of the NHC ring, whereas the yz plane is the plane orthogonal to the mean plane of the NHC ring, and passing through the carbene C atom of the NHC ring. The metal is at the origin. S11

Table S8. Steric maps for COOtBu-alkyl (radius of 5 Å). The isocontour curves of the steric maps are in Å. The xz plane is the mean plane of the NHC ring, whereas the yz plane is the plane orthogonal to the mean plane of the NHC ring, and passing through the carbene C atom of the NHC ring. The metal is at the origin. S15

Table S9. Steric maps for COOtBu-cph3 (radius of 5 Å). The isocontour curves of the steric maps are in Å. The xz plane is the mean plane of the NHC ring, whereas the yz plane is the plane orthogonal to the mean plane of the NHC ring, and passing through the carbene C atom of the NHC ring. The metal is at the origin. S19

Table S10. Steric maps for alkyl-COOtBu (radius of 5 Å). The isocontour curves of the steric maps are in Å. The xz plane is the mean plane of the NHC ring, whereas the yz plane is the plane orthogonal to the mean plane of the NHC ring, and passing through the carbene C atom of the NHC ring. The metal is at the origin. S23

Table S11. Correlation between %V_{Bur} and Energies (relative Gibbs energies in kcal/mol)....S25

Figure S1. 2D NCI Plots, and correlations. S27

Figure S2. Correlations between relative energies in kcal/mol and %V_{Bur}. S28

Figure S3. Correlations between relative energies in kcal/mol and %V_{Bur}. S31

Energy Calculation Tables

Table S1. Conformational search and preoptimization for system **1** (via CREST + GAUSSIAN09), absolute energies in a.u. Initial geometry obtained from optimization of the XRD precatalyst structure upon removing the chelate chain of the ylidene.

	Conformational system 1							
	E_{gas}	ΔE_{gas}	G_{gas}	ΔG_{gas}	E_{sol}	ΔE_{sol}	ΔG_{sol}	ΔG_{sol}
**/initial geom/**	-3668.74603206	0.00	-3667.871032	0.00	-3669.46364623	0.00	-3668.58864617	**0.00**
**cat-conf1**	Non Converged	------	------	------	------	------	------	**------**
**cat-conf2**	Non Converged	------	------	------	------	------	------	**------**
**cat-conf3**	Non Converged	------	------	------	------	------	------	**------**
**cat-conf4**	-3816.67517744	0.20	-3815.783169	-1.45	-3817.49438515	-0.01	-3816.60237671	**-1.67**
**cat-conf5**	-3816.67517728	0.20	-3815.783179	-1.46	-3817.49438569	-0.01	-3816.60238741	**-1.67**
**cat-conf6**	-3816.66923140	3.93	-3815.775246	3.52	-3817.49012379	2.66	-3816.59613839	**2.25**
**cat-conf7**	-3816.66923157	3.93	-3815.775266	3.51	-3817.49011994	2.66	-3816.59615437	**2.24**
**cat-conf8**	-3816.66923150	3.93	-3815.775271	3.50	-3817.49012041	2.66	-3816.59615991	**2.23**
**cat-conf9**	-3816.67582098	-0.20	-3815.780454	0.25	-3817.49035594	2.52	-3816.59498896	**2.97**
**cat-confopt**	-3816.67397585	0.96	-3815.779767	0.68	-3817.49272324	1.03	-3816.59851439	**0.76**

Table S2. Conformational search and preoptimization for system **2** (via CREST + GAUSSIAN09), absolute energies in a.u. Initial geometry obtained from optimization of the XRD precatalyst structure upon removing the chelate chain of the ylidene.

	Conformational system 2							
	E_{gas}	ΔE_{gas}	G_{gas}	ΔG_{gas}	E_{sol}	ΔE_{sol}	ΔG_{sol}	ΔG_{sol}
**/initial geom/**	-3668.74603206	0.00	-3667.871032	0.00	-3669.46364623	0.00	-3668.58864617	**0.00**
**neuch2-conf1**	-3668.75082315	-3.01	-3.667.87800	-4.37	-3669.46858806	-3.10	-3668.59576391	**-4.47**
**neuch2-conf2**	-3668.75082279	-3.01	-3.667.87802	-4.38	-3669.46860630	-3.11	-3668.59580051	**-4.49**
**neuch2-conf3**	-3668.74777016	-1.09	-3.667.87459	-2.23	-3669.46472567	-0.68	-3668.59154751	**-1.82**
**neuch2-conf4**	-3668.74726212	-0.77	-3.667.87513	-2.57	-3669.46449918	-0.54	-3668.59236506	**-2.33**
**neuch2-conf5**	-3668.74366435	1.49	-3.667.87071	0.20	-3669.46165629	1.25	-3668.58870294	**-0.04**
**neuch2-conf6**	-3668.74726207	-0.77	-3.667.87512	-2.57	-3669.46450282	-0.54	-3668.59236475	**-2.33**
**neuch2-conf7**	-3668.74765956	-1.02	-3.667.87451	-2.18	-3669.46260348	0.65	-3668.58944992	**-0.50**
**neuch2-conf8**	-3668.75011023	-2.56	-3.667.87864	-4.77	-3669.46917765	-3.47	-3668.59770642	**-5.69**
**neuch2-conf9**	-3668.74670327	-0.42	-3.667.87228	-0.78	-3669.45776722	3.69	-3668.58333995	**3.33**

Table S3. Precatalyst path optimization for system **1** (GAUSSIAN09), absolute energies in a.u.

	System 1							
	E_{gas}	ΔE_{gas}	G_{gas}	ΔG_{gas}	E_{sol}	ΔE_{sol}	ΔG_{sol}	ΔG_{sol}
**precat**	-4240.68238704	0.0	-4.239.624734	0.0	-4.241.60007832	0.0	-4.240.54242528	**0.0**
**TS0**	-4240.63793036	27.9	-4.239.582270	26.6	-4.241.55984301	25.2	-4.240.50418265	**24.0**
**14e'**	-4240.64449233	23.8	-4.239.591273	21.0	-4.241.57547915	15.4	-4.240.52225982	**12.7**
**CI1'**	-4319.20210743	3.7	-4.318.098086	15.8	-4.320.14248987	3.4	-4.319.03846844	**15.5**
**TS1'**	-4319.19465302	8.4	-4.318.087943	22.2	-4.320.13966383	5.2	-4.319.03295381	**19.0**
**MCB'**	-4319.20546339	1.6	-4.318.095879	17.2	-4.320.15425182	-4.0	-4.319.04466743	**11.6**
**TS2'**	-4319.18908826	11.9	-4.318.082839	25.4	-4.320.13789148	6.3	-4.319.03164222	**19.8**
**CI2'**	-4319.18850679	12.2	-4.318.084932	24.1	-4.320.13795136	6.2	-4.319.03437657	**18.1**
14e'	-3816.67550122	48.0	-3.815.780855	39.7	-3.817.49436446	24.5	-3.816.59971824	**16.1**

Table S4. Precatalyst path optimization for system **2** (GAUSSIAN09), absolute energies in a.u.

	System 2							
	E_{gas}	ΔE_{gas}	G_{gas}	ΔG_{gas}	E_{sol}	ΔE_{sol}	ΔG_{sol}	ΔG_{sol}
**precat**	-4092.74036141	0.0	-4091.702336	0.0	-4093.56302478	0.0	-4092.52499937	0.0
**TS0**	-4092.68734221	33.3	-4091.655052	29.7	-4093.51563531	29.7	-4092.48334510	26.1
**14e'**	-4092.68734221	22.5	-4091.678521	14.9	-4093.54131736	13.6	-4092.51537571	6.0
**CI1'**	-4092.70446265	1.4	-4170.177843	14.5	-4172.10287392	5.0	-4171.01692972	18.1
**TS1'**	-4171.2604290	3.5	-4170.177055	15.0	-4172.10372253	4.5	-4171.02034853	16.0
**MCB'**	-4171.26895105	-1.9	-4170.182790	11.4	-4172.11639377	-3.5	-4171.03023272	9.8
**TS2'**	-4171.24437988	13.5	-4170.163386	23.5	-4172.10195714	5.6	-4171.02096326	15.6
**CI2'**								
14e'	-3668.75011023	-2.56	-3.667.87864	-4.77	-3669.46917765	-3.47	-3668.59770642	**6.6**

Table S5. Precatalyst path optimization for system **3** (GAUSSIAN09), absolute energies in a.u.

	System 3							
	E_{gas}	ΔE_{gas}	G_{gas}	ΔG_{gas}	E_{sol}	ΔE_{sol}	ΔG_{sol}	ΔG_{sol}
**precat**	-2403,38552315	0.0	-2402.854908	0.0	-2403.77332721	0.0	-2403.24271206	**0.0**
**TS0**	-2403.348309	23.4	-2402.817831	23.3	-2403.73939377	21.3	-2403.208915	**21.2**
**14e'**	-2403.35922248	16.5	-2402.834228	13.0	-2403.75732591	10.0	-2403.23233143	**6.5**
**CI1'**	-2481.91806201	-4.4	-2481.340100	8.4	-2482.32314412	-1.3	-2481.74518211	**11.5**
**TS1'**	-2481.90925790	1.2	-2481.331973	13.5	-2482.32105984	0.0	-2481.74377494	**12.4**
**MCB'**	-2481.91881485	-4.8	-2481.338267	9.5	-2482.33332949	-7.7	-2481.75278164	**6.7**
**TS2'**	-2481.90072415	6.5	-2481.323433	18.8	-2482.31380293	4.6	-2481.73651178	**16.9**
**CI2'**	-2481.90597223	3.2	-2481.329214	15.2	-2482.31769691	2.2	-2481.74093868	**14.2**
14e'	-1979.41053194	28.0	-1979.039484	21.8	-1979.67912875	17.2	-1979.30808081	**11.1**

There is a summary of relative energies in kcal/mol in Table S6.

Table S6. Summary of the relative energies for the IN and OUT conformations (GAUSSIAN09), in kcal/mol.

	**Egas**	**Ggas**	**Esol**	**Gsol**
**IN-*cis***	-44.07	-15.46	-13.98	14.63
**IN-*trans***	-52.23	-24.41	-19.53	8.28
**OUT-*cis***	-42.11	-16.45	-17.96	7.69
**OUT-*trans***	-40.42	-15.58	-23.73	1.11
	NHC	olefin		
**IN-*cis***	21.32	5.85		
**IN-*trans***	21.27	5.89		
**OUT-*cis***	21.64	5.89		
**OUT-*trans***	21.53	5.88		
	**Egas**	**Ggas**	**Esol**	**Gsol**
**IN-*cis***	-57.06	-25.4	-13.76	17.86
**IN-*trans***	-61.77	-33.06	-26.28	2.43
**OUT-*cis***	-47.96	-19.99	-15.07	12.90
**OUT-*trans***	-46.54	-17.98	-13.38	15.18
	NHC	olefin		
**IN-*cis***	21.37	10.84		
**IN-*trans***	21.04	10.99		
**OUT-*cis***	21.42	11.06		
**OUT-*trans***	21.39	10.88		

Steric maps

Table S7. Steric maps for COOtBu-alkyl (radius of 10 Å). The isocontour curves of the steric maps are in Å. The xz plane is the mean plane of the NHC ring, whereas the yz plane is the plane orthogonal to the mean plane of the NHC ring, and passing through the carbene C atom of the NHC ring. The metal is at the origin.

system 1 - MCB-IN*cis*_COOtBu-alkyl
NHC (no Hs)

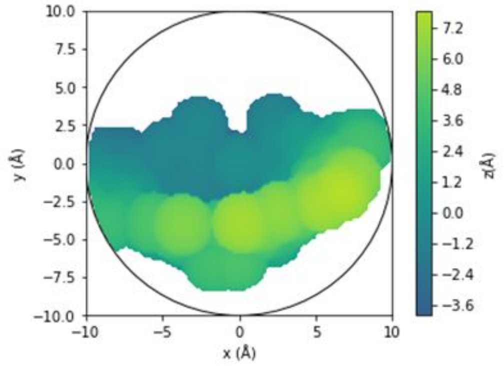

**Center:**	92	**NE** 14.50<br>**NW** 9.87<br>**SE** 33.29<br>**SW** 27.61<br>**Total** 21.32
**Z_axis**	6;10	
**xz_planes**	10	
**density**	0.0001	
**excluded atoms**	1,2,3,17,133,134,135,136,137,138,139,140,141,142,143,144,145,146,147,148,149,127,131,150,132,151,152,153,154,155,156,157,158,159,160	

system 1 - MCB-IN*cis*_COOtBu-alkyl
olefin (no Hs)

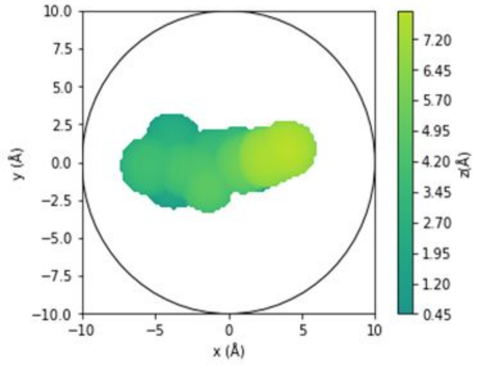

**Center:**	92	**NE** 6.28<br>**NW** 5.59<br>**SE** 7.35<br>**SW** 4.17<br>**Total** 5.85
**Z_axis**	6;10	
**xz_planes**	10	
**density**	0.0001	
**excluded atoms**	1,2,3,4, 5, 6, 7, 8, 9, 10, 11, 12, 13, 14, 15, 16, 18, 19, 20, 21, 22, 23, 24, 25, 26, 27, 28, 29, 30, 31, 32, 33, 34, 35, 36, 37, 38, 39, 40, 41, 42, 43, 44, 45, 46, 47, 48, 49, 50, 51, 52, 53, 54, 55, 56, 57, 58, 59, 60, 61, 62, 63, 64, 65, 66, 67, 68, 69, 70, 71, 72, 73, 74, 75, 76,	

	77, 78, 79, 80, 81, 82, 83, 84, 85, 86, 87, 88, 89, 90, 91, 92, 93, 94, 95, 96, 97, 98, 99, 100, 101, 102, 103, 104, 105, 106, 107, 108, 109, 110, 111, 112, 113, 114, 115, 116, 117, 118, 119, 120, 121, 122, 123, 124, 125

system 1 - MCB-IN*trans*_COOtBu-alkyl
NHC (no Hs)

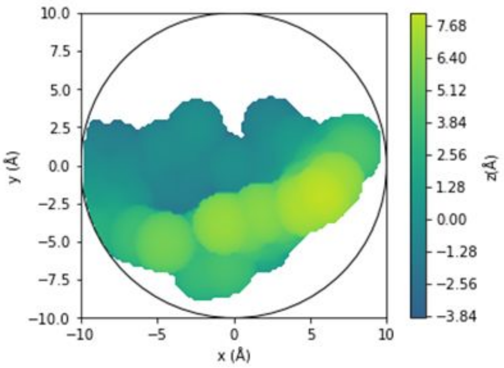

**Center:**	92	**NE** 13.79
**Z_axis**	6;10	**NW** 10.53
**xz_planes**	10	**SE** 33.50
**density**	0.0001	**SW** 27.24<br>**Total** 21.27
**excluded atoms**	[1,2,3,17,134,135,136,137,138,139,140,141,142,143,144,145,146,147,148,149,150,127,128,131,132,133,151,152,153,154,155,156,157,158,159,160,126,129,130]	

system 1 - MCB-IN*trans*_COOtBu-alkyl
olefin (no Hs)

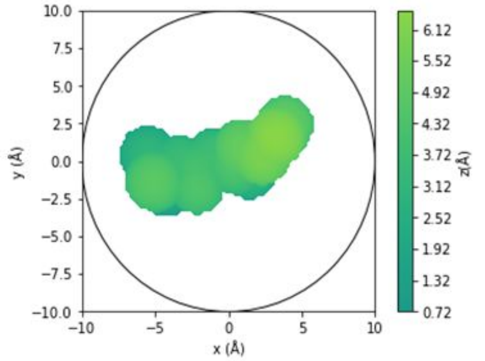

**Center:**	92	**NE** 7.14
**Z_axis**	6;10	**NW** 4.36
**xz_planes**	10	**SE** 8.80
**density**	0.0001	**SW** 3.26<br>**Total** 5.89
**excluded atoms**	1,2,3,4, 5, 6, 7, 8, 9, 10, 11, 12, 13, 14, 15, 16, 18, 19, 20, 21, 22, 23, 24, 25, 26, 27, 28, 29, 30, 31, 32, 33, 34, 35, 36, 37, 38, 39, 40, 41, 42, 43, 44, 45, 46, 47, 48, 49, 50, 51, 52, 53, 54, 55, 56, 57, 58, 59, 60, 61, 62, 63, 64, 65, 66, 67, 68, 69, 70, 71, 72, 73, 74, 75, 76, 77, 78, 79, 80, 81, 82, 83, 84, 85, 86, 87, 88, 89, 90, 91, 92, 93, 94, 95, 96, 97, 98, 99, 100, 101, 102, 103, 104, 105, 106, 107, 108, 109, 110, 111, 112, 113, 114, 115, 116, 117, 118, 119, 120, 121, 122, 123, 124, 125	

system 1 - MCB-OUT*cis*_COOtBu-alkyl
NHC (no Hs)

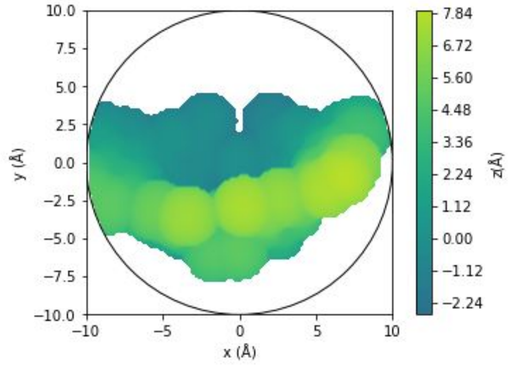

**Center:**	92	**NE** 16.90<br>**NW** 13.36<br>**SE** 30.91<br>**SW** 25.39<br>**Total** 21.64
**Z_axis**	6;10	
**xz_planes**	10	
**density**	0.0001	
**excluded atoms**	[1,2,3,17,131,132,133,134,135,136,137,138,139,140,141,142,143,144,145,146,147,127,128,149,148,150,151,152,153,155,154,156,158,157,159,160,126,129,130]	

system 1 - MCB-OUT*cis*_COOtBu-alkyl
olefin (no Hs)

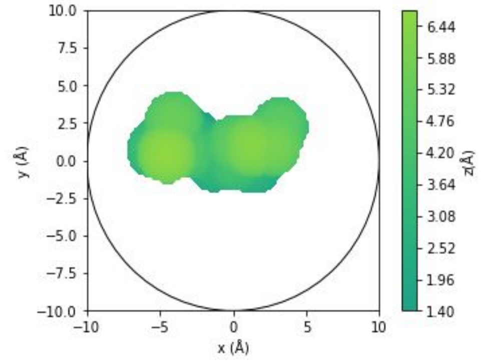

**Center:**	92	**NE** 6.75<br>**NW** 10.40<br>**SE** 3.73<br>**SW** 2.66<br>**Total** 5.89
**Z_axis**	6;10	
**xz_planes**	10	
**density**	0.0001	
**excluded atoms**	1,2,3,4, 5, 6, 7, 8, 9, 10, 11, 12, 13, 14, 15, 16, 18, 19, 20, 21, 22, 23, 24, 25, 26, 27, 28, 29, 30, 31, 32, 33, 34, 35, 36, 37, 38, 39, 40, 41, 42, 43, 44, 45, 46, 47, 48, 49, 50, 51, 52, 53, 54, 55, 56, 57, 58, 59, 60, 61, 62, 63, 64, 65, 66, 67, 68, 69, 70, 71, 72, 73, 74, 75, 76, 77, 78, 79, 80, 81, 82, 83, 84, 85, 86, 87, 88, 89, 90, 91, 92, 93, 94, 95, 96, 97, 98, 99, 100, 101, 102, 103, 104, 105, 106, 107, 108, 109, 110, 111, 112, 113, 114, 115, 116, 117, 118, 119, 120, 121, 122, 123, 124, 125	

system 1 - MCB-OUT*trans*_COOtBu-alkyl
NHC (no Hs)

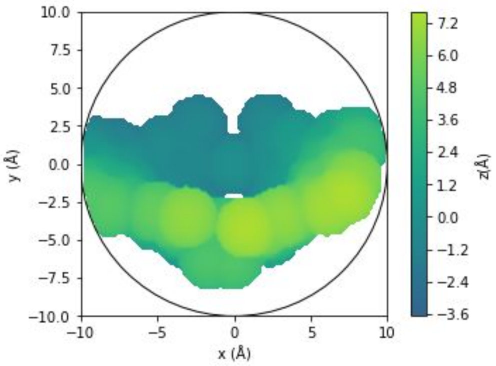

**Center:**	92	**NE** 14.76<br>**NW** 11.51<br>**SE** 32.06<br>**SW** 27.81<br>**Total** 21.53
**Z_axis**	6;10	
**xz_planes**	10	
**density**	0.0001	
**excluded atoms**	1,2,3,17,131,132,133,134,135,136,137,138,139,140,141,142,143,144,145,146,147,127,128,149,148,150,152,151,153,155,154,156,157,158,158,160,126,129,130	

system 1 - MCB-OUT*trans*_COOtBu-alkyl
olefin (no Hs)

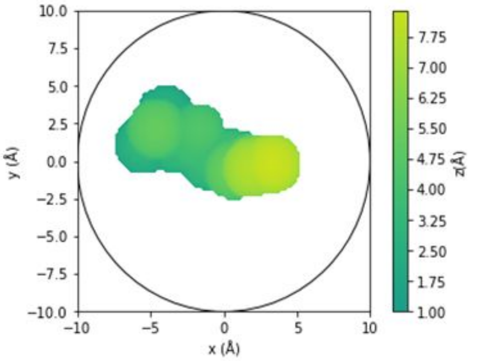

**Center:**	92	**NE** 4.34<br>**NW** 11.15<br>**SE** 2.30<br>**SW** 5.72<br>**Total** 5.88
**Z_axis**	6;10	
**xz_planes**	10	
**density**	0.0001	
**excluded atoms**	1,2,3,4, 5, 6, 7, 8, 9, 10, 11, 12, 13, 14, 15, 16, 18, 19, 20, 21, 22, 23, 24, 25, 26, 27, 28, 29, 30, 31, 32, 33, 34, 35, 36, 37, 38, 39, 40, 41, 42, 43, 44, 45, 46, 47, 48, 49, 50, 51, 52, 53, 54, 55, 56, 57, 58, 59, 60, 61, 62, 63, 64, 65, 66, 67, 68, 69, 70, 71, 72, 73, 74, 75, 76, 77, 78, 79, 80, 81, 82, 83, 84, 85, 86, 87, 88, 89, 90, 91, 92, 93, 94, 95, 96, 97, 98, 99, 100, 101, 102, 103, 104, 105, 106, 107, 108, 109, 110, 111, 112, 113, 114, 115, 116, 117, 118, 119, 120, 121, 122, 123, 124, 125	

Table S8. Steric maps for COOtBu-cph3 (radius of 10 Å). The isocontour curves of the steric maps are in Å. The xz plane is the mean plane of the NHC ring, whereas the yz plane is the plane orthogonal to the mean plane of the NHC ring, and passing through the carbene C atom of the NHC ring. The metal is at the origin.

System 1 – MCB-In*cis*_COOtBu-cph3
NHC (no Hs)

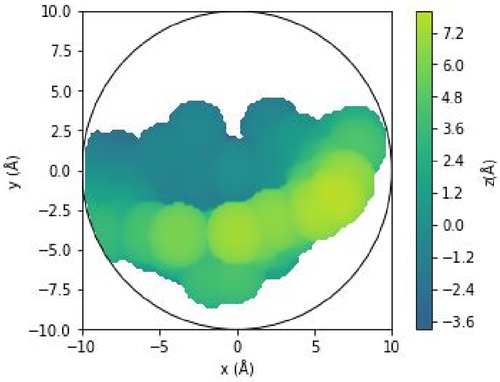

**Center:**	92	**NE** 15.55<br>**NW** 9.89<br>**SE** 33.40<br>**SW** 26.62<br>**Total** 21.37
**Z_axis**	6;10	
**xz_planes**	10	
**density**	0.0001	
**excluded atoms**	1,2,3,17,134,146,147,148,149,150,151,152,153,154,155,156,157,158,159,160,161,128,127,131,132,162,133,135,136,137,138,139,140,141,142,143,144,145,163,164,165,166,167,168,169,170,171,172,173,174,175,176,177,178,179,180,181,182,183,184,126,129,130	

system 1 – MCB-IN*cis*_COOtBu-alkyl
olefin (no Hs)

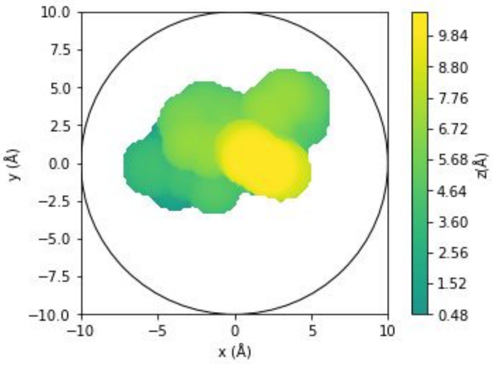

**Center:**	92	**NE** 15.74<br>**NW** 13.09<br>**SE** 8.02<br>**SW** 6.53
**Z_axis**	6;10	

**xz_ planes**	10	**Total** 10.84
**den sity**	0.0001	
**excl ude d ato ms**	1,2,3, 4, 5, 6, 7, 8, 9, 10, 11, 12, 13, 14, 15, 16, 18, 19, 20, 21, 22, 23, 24, 25, 26, 27, 28, 29, 30, 31, 32, 33, 34, 35, 36, 37, 38, 39, 40, 41, 42, 43, 44, 45, 46, 47, 48, 49, 50, 51, 52, 53, 54, 55, 56, 57, 58, 59, 60, 61, 62, 63, 64, 65, 66, 67, 68, 69, 70, 71, 72, 73, 74, 75, 76, 77, 78, 79, 80, 81, 82, 83, 84, 85, 86, 87, 88, 89, 90, 91, 92, 93, 94, 95, 96, 97, 98, 99, 100, 101, 102, 103, 104, 105, 106, 107, 108, 109, 110, 111, 112, 113, 114, 115, 116, 117, 118, 119, 120, 121, 122, 123, 124, 125	

system 1 - MCB-IN*trans*_COOtBu-cph3
NHC (no Hs)

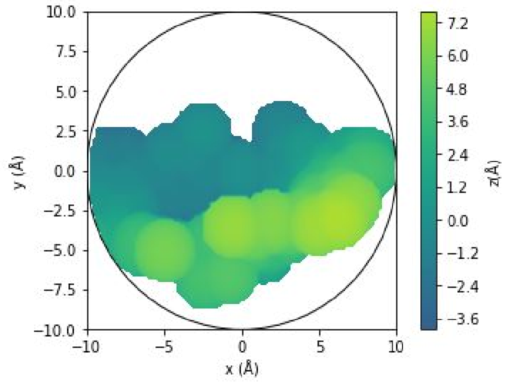

**Center:**	92	**NE** 11.85
**Z_axis**	6;10	**NW** 10.53
**xz_planes**	10	**SE** 33.50
**density**	0.0001	**SW** 27.24
		**Total** 21.27
**excluded atoms**	1,2,3,17,135,147,148,149,150,151,152,153,154,155,156,157,158,159,160,161,162,127,128,131,132,133,134,136,137,138,139,140,141,142,143,144,145,146,163,164,165,166,167,168,169,170,171,172,173,126,129,130,174,175,176,177,178,179,180,181,182,183,184	

system 1 - MCB-IN*trans*_COOtBu-cph3
olefin (no Hs)

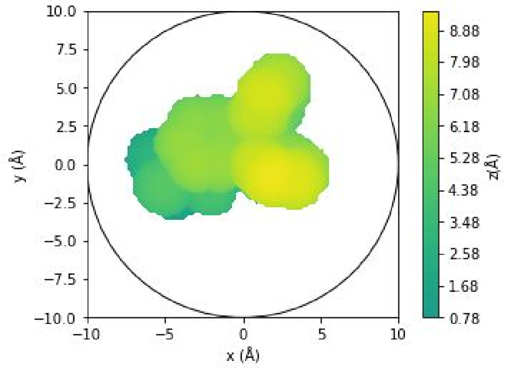

**Center:**	92	**NE** 14.09
**Z_axis**	6;10	**NW** 11.65
**xz_planes**	10	**SE** 10.49
**density**	0.0001	**SW** 7.73
		**Total** 14.09
**excluded atoms**	1,2,3,4, 5, 6, 7, 8, 9, 10, 11, 12, 13, 14, 15, 16, 18, 19, 20, 21, 22, 23, 24, 25, 26, 27, 28, 29, 30, 31, 32, 33, 34, 35, 36, 37, 38, 39, 40, 41, 42, 43, 44, 45, 46, 47, 48, 49, 50, 51, 52, 53, 54, 55, 56, 57, 58, 59, 60, 61, 62, 63, 64, 65, 66, 67, 68, 69, 70, 71, 72, 73, 74, 75, 76, 77, 78, 79, 80, 81, 82, 83, 84, 85, 86, 87, 88, 89, 90, 91, 92, 93, 94, 95, 96, 97, 98, 99, 100, 101, 102, 103, 104, 105, 106, 107, 108, 109, 110, 111, 112, 113, 114, 115, 116, 117, 118, 119, 120, 121, 122, 123, 124, 125	

system 1 - MCB-OUT*cis*_COOtBu-cph3<br>**NHC (no Hs)**		
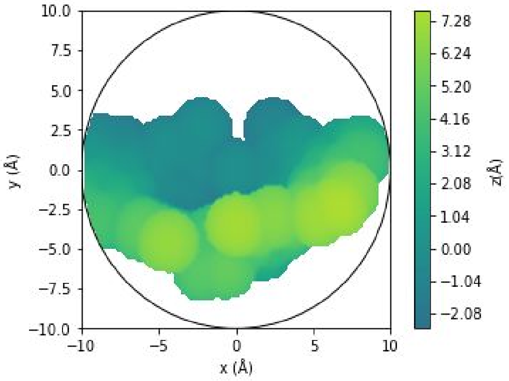		
**Center:**	92	**NE** 13.48<br>**NW** 13.36<br>**SE** 30.91<br>**SW** 25.39<br>**Total** 21.64
**Z_axis**	6;10	
**xz_planes**	10	
**density**	0.0001	
**excluded atoms**	1,2,3,17,135,147,148,149,150,151,152,153,154,155,156,157,158,159,160,161,162,127,128,131,132,133,134,136,137,138,139,140,141,142,143,144,145,146,163,164,165,166,167,168,169,170,171,172,173,126,129,130,174,175,176,177,178,179,180,181,182,183,184	

system 1 - MCB-OUT*cis*_COOtBu-cph3<br>**olefin (no Hs)**		
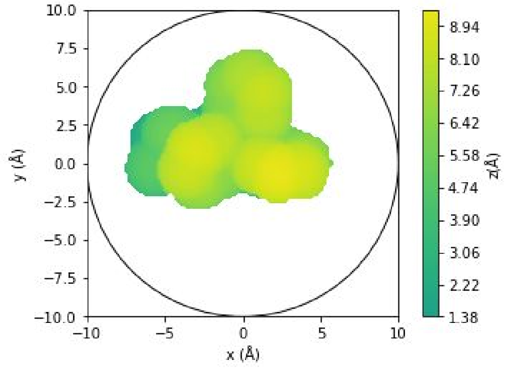		
**Center:**	92	**NE** 11.45<br>**NW** 17.57<br>**SE** 8.39<br>**SW** 6.83<br>**Total** 11.06
**Z_axis**	6;10	
**xz_planes**	10	
**density**	0.0001	
**excluded atoms**	1,2,3,4, 5, 6, 7, 8, 9, 10, 11, 12, 13, 14, 15, 16, 18, 19, 20, 21, 22, 23, 24, 25, 26, 27, 28, 29, 30, 31, 32, 33, 34, 35, 36, 37, 38, 39, 40, 41, 42, 43, 44, 45, 46, 47, 48, 49, 50, 51, 52, 53, 54, 55, 56, 57, 58, 59, 60, 61, 62, 63, 64, 65, 66, 67, 68, 69, 70, 71, 72, 73, 74, 75, 76, 77, 78, 79, 80, 81, 82, 83, 84, 85, 86, 87, 88, 89, 90, 91, 92, 93, 94, 95, 96, 97, 98, 99, 100, 101, 102, 103, 104, 105, 106, 107, 108, 109, 110, 111, 112, 113, 114, 115, 116, 117, 118, 119, 120, 121, 122, 123, 124, 125	

system 1 - MCB-OUT*trans*_COOtBu-cph3<br>**NHC (no Hs)**

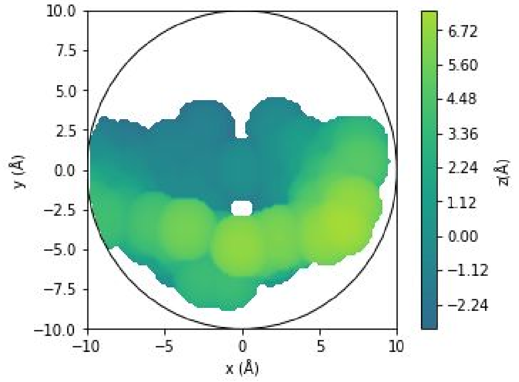

**Center:**	92	**NE**	13.22
**Z_axis**	6;10	**NW**	11.35
**xz_planes**	10	**SE**	33.09
**density**	0.0001	**SW**	27.89
		**Total**	13.22
**excluded atoms**	1,2,3,17,135,147,148,149,150,151,152,153,154,155,156,157,158,159,160,161,162,127,128,131,132,133,134,136,137,138,139,140,141,142,143,144,145,146,163,164,165,166,167,168,169,170,171,172,173,126,129,130,174,175,176,177,178,179,180,181,182,183,184		

system 1 - MCB-OUT*trans*_COOtBu-cph3
olefin (no Hs)

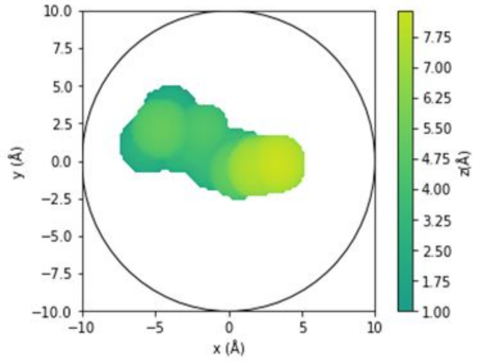

**Center:**	92	**NE** 11.51
**Z_axis**	6;10	**NW** 14.79
**xz_planes**	10	**SE** 6.52
**density**	0.0001	**SW** 10.71
		**Total** 10.88
**excluded atoms**	1,2,3,4, 5, 6, 7, 8, 9, 10, 11, 12, 13, 14, 15, 16, 18, 19, 20, 21, 22, 23, 24, 25, 26, 27, 28, 29, 30, 31, 32, 33, 34, 35, 36, 37, 38, 39, 40, 41, 42, 43, 44, 45, 46, 47, 48, 49, 50, 51, 52, 53, 54, 55, 56, 57, 58, 59, 60, 61, 62, 63, 64, 65, 66, 67, 68, 69, 70, 71, 72, 73, 74, 75, 76, 77, 78, 79, 80, 81, 82, 83, 84, 85, 86, 87, 88, 89, 90, 91, 92, 93, 94, 95, 96, 97, 98, 99, 100, 101, 102, 103, 104, 105, 106, 107, 108, 109, 110, 111, 112, 113, 114, 115, 116, 117, 118, 119, 120, 121, 122, 123, 124, 125	

The sensibility towards steric changes is lost with the analysis radius of 10.0 Å. Consequently, it was repeated with radius 5.0 Å.

Table S8. Steric maps for COOtBu-alkyl (radius of 5 Å). The isocontour curves of the steric maps are in Å. The xz plane is the mean plane of the NHC ring, whereas the yz plane is the plane orthogonal to the mean plane of the NHC ring, and passing through the carbene C atom of the NHC ring. The metal is at the origin.

system 1 - MCB-IN*cis*_COOtBu-alkyl<br>**NHC (no Hs)**		
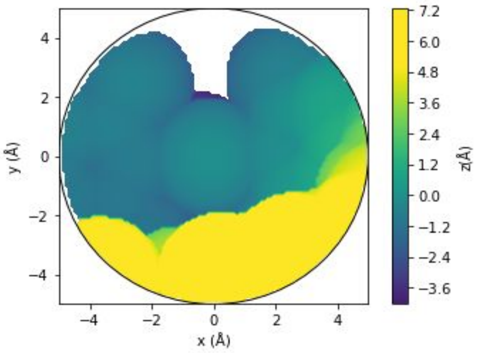		
**Center:**	92	**NE** 42.70<br>**NW** 31.79<br>**SE** 42.80<br>**SW** 62.05<br>**Total** 44.83
**Z_axis**	6;10	
**xz_planes**	10	
**density**	0.0001	
**excluded atoms**	1,2,3,17,133,134,135,136,137,138,139,140,141,142,143,144,145,146,147,148,149,127,131,150,132,151,152,153,154,155,156,157,158,159,160	

system 1 - MCB-IN*cis*_COOtBu-alkyl<br>**olefin (no Hs)**		
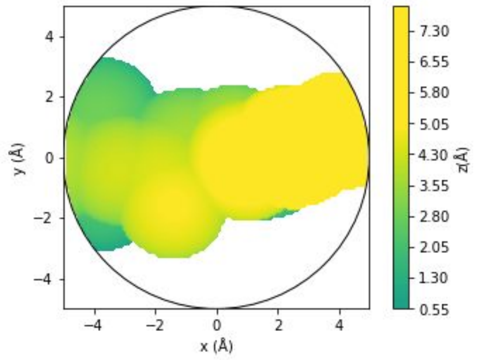		
**Center:**	92	**NE** 23.04<br>**NW** 31.79<br>**SE** 40.80<br>**SW** 21.61<br>**Total** 29.31
**Z_axis**	6;10	
**xz_planes**	10	
**density**	0.0001	
**excluded atoms**	1,2,3,4, 5, 6, 7, 8, 9, 10, 11, 12, 13, 14, 15, 16, 18, 19, 20, 21, 22, 23, 24, 25, 26, 27, 28, 29, 30, 31, 32, 33, 34, 35, 36, 37, 38, 39, 40, 41, 42, 43, 44, 45, 46, 47, 48, 49, 50, 51, 52, 53, 54, 55, 56, 57, 58, 59, 60, 61, 62, 63, 64, 65, 66, 67, 68, 69, 70, 71, 72, 73, 74, 75, 76, 77, 78, 79, 80, 81, 82, 83, 84, 85, 86, 87, 88, 89, 90, 91, 92, 93, 94, 95, 96, 97, 98, 99, 100, 101, 102, 103, 104, 105, 106, 107, 108, 109, 110, 111, 112, 113, 114, 115, 116, 117, 118, 119, 120, 121, 122, 123, 124, 125	

system 1 - MCB-IN*trans*_COOtBu-alkyl<br>**NHC (no Hs)**

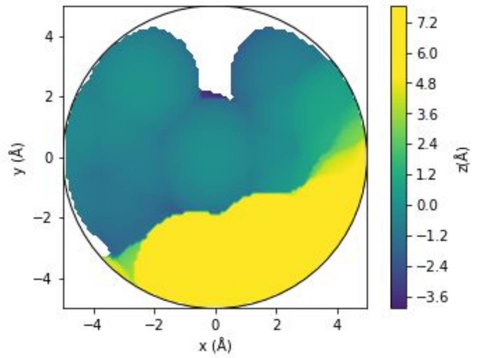

**Center:**	92	**NE** 41.87
**Z_axis**	6;10	**NW** 37.20
**xz_planes**	10	**SE** 39.85
**density**	0.0001	**SW** 67.33<br>**Total** 46.56
**excluded atoms**	[1,2,3,17,134,135,136,137,138,139,140,141,142,143,144,145,146,147,148,149,150,127,128,131,132,133,151,152,153,154,155,156,157,158,159,160,126,129,130]	

system 1 - MCB-IN*trans*_COOtBu-alkyl
olefin (no Hs)

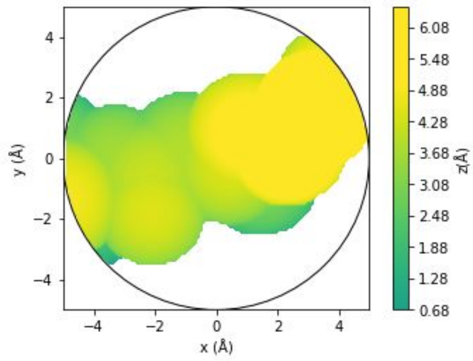

**Center:**	92	**NE** 24.69
**Z_axis**	6;10	**NW** 16.69
**xz_planes**	10	**SE** 37.26
**density**	0.0001	**SW** 22.82<br>**Total** 25.37
**excluded atoms**	1,2,3,4, 5, 6, 7, 8, 9, 10, 11, 12, 13, 14, 15, 16, 18, 19, 20, 21, 22, 23, 24, 25, 26, 27, 28, 29, 30, 31, 32, 33, 34, 35, 36, 37, 38, 39, 40, 41, 42, 43, 44, 45, 46, 47, 48, 49, 50, 51, 52, 53, 54, 55, 56, 57, 58, 59, 60, 61, 62, 63, 64, 65, 66, 67, 68, 69, 70, 71, 72, 73, 74, 75, 76, 77, 78, 79, 80, 81, 82, 83, 84, 85, 86, 87, 88, 89, 90, 91, 92, 93, 94, 95, 96, 97, 98, 99, 100, 101, 102, 103, 104, 105, 106, 107, 108, 109, 110, 111, 112, 113, 114, 115, 116, 117, 118, 119, 120, 121, 122, 123, 124, 125	

system 1 - MCB-OUT*cis*_COOtBu-alkyl
NHC (no Hs)

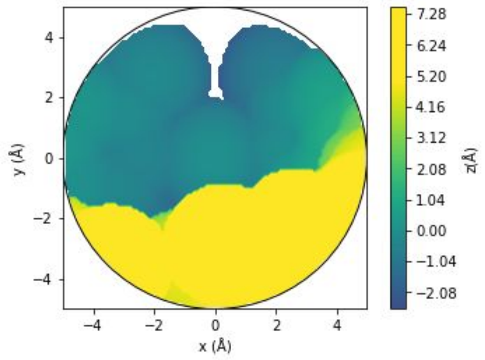

**Center:**	92	**NE** 44.75

**Z_axis**	6;10	**NW** 40.15
**xz_planes**	10	**SE** 50.19
**density**	0.0001	**SW** 64.59
		**Total** 49.92
**excluded atoms**	[1,2,3,17,131,132,133,134,135,136,137,138,139,140,141,142,143,144,145,146,147,127,128,149,148,150,151,152,153,155,154,156,158,157,159,160,126,129,130]	

system 1 - MCB-OUT*cis*_COOtBu-alkyl
olefin (no Hs)

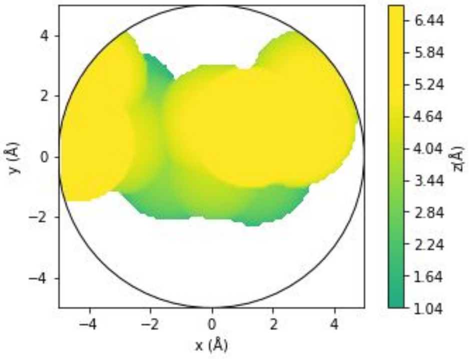

**Center:**	92	**NE** 28.27
**Z_axis**	6;10	**NW** 37.83
**xz_planes**	10	**SE** 17.77
**density**	0.0001	**SW** 18.78
		**Total** 25.67
**excluded atoms**	1,2,3,4, 5, 6, 7, 8, 9, 10, 11, 12, 13, 14, 15, 16, 18, 19, 20, 21, 22, 23, 24, 25, 26, 27, 28, 29, 30, 31, 32, 33, 34, 35, 36, 37, 38, 39, 40, 41, 42, 43, 44, 45, 46, 47, 48, 49, 50, 51, 52, 53, 54, 55, 56, 57, 58, 59, 60, 61, 62, 63, 64, 65, 66, 67, 68, 69, 70, 71, 72, 73, 74, 75, 76, 77, 78, 79, 80, 81, 82, 83, 84, 85, 86, 87, 88, 89, 90, 91, 92, 93, 94, 95, 96, 97, 98, 99, 100, 101, 102, 103, 104, 105, 106, 107, 108, 109, 110, 111, 112, 113, 114, 115, 116, 117, 118, 119, 120, 121, 122, 123, 124, 125	

system 1 - MCB-OUT*trans*_COOtBu-alkyl
NHC (no Hs)

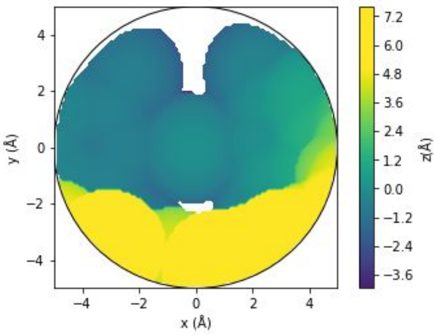

**Center:**	92	**NE** 44.55
**Z_axis**	6;10	**NW** 33.18
**xz_planes**	10	**SE** 48.36
**density**	0.0001	**SW** 58.73
		**Total** 46.20
**excluded atoms**	1,2,3,17,131,132,133,134,135,136,137,138,139,140,141,142,143,144,145,146,147,127,128,149,148,150,152,151,153,155,154,156,157,158,158,160,126,129,130	

system 1 - MCB-OUT*trans*_COOtBu-alkyl
olefin (no Hs)

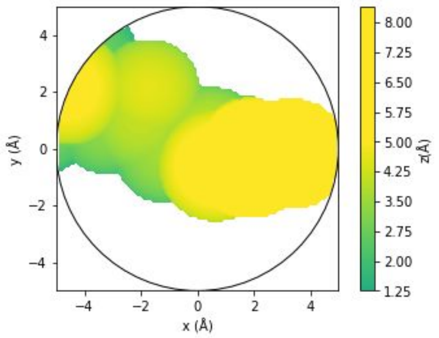

**Center:**	92	**NE** 19.41
**Z_axis**	6;10	**NW** 40.81
**xz_planes**	10	**SE** 15.69
**density**	0.0001	**SW** 22.56<br>**Total** 24.62
**excluded atoms**	1,2,3,4, 5, 6, 7, 8, 9, 10, 11, 12, 13, 14, 15, 16, 18, 19, 20, 21, 22, 23, 24, 25, 26, 27, 28, 29, 30, 31, 32, 33, 34, 35, 36, 37, 38, 39, 40, 41, 42, 43, 44, 45, 46, 47, 48, 49, 50, 51, 52, 53, 54, 55, 56, 57, 58, 59, 60, 61, 62, 63, 64, 65, 66, 67, 68, 69, 70, 71, 72, 73, 74, 75, 76, 77, 78, 79, 80, 81, 82, 83, 84, 85, 86, 87, 88, 89, 90, 91, 92, 93, 94, 95, 96, 97, 98, 99, 100, 101, 102, 103, 104, 105, 106, 107, 108, 109, 110, 111, 112, 113, 114, 115, 116, 117, 118, 119, 120, 121, 122, 123, 124, 125	

Table S9. Steric maps for COOtBu-cph3 (radius of 5 Å). The isocontour curves of the steric maps are in Å. The xz plane is the mean plane of the NHC ring, whereas the yz plane is the plane orthogonal to the mean plane of the NHC ring, and passing through the carbene C atom of the NHC ring. The metal is at the origin.

system 1 - MCB-IN*cis*_COOtBu-cph3
NHC (no Hs)

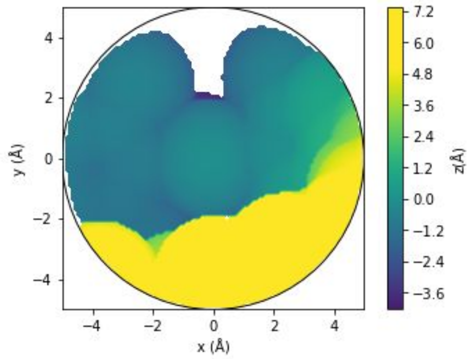

**Center:**	92	NE 44.75<br>NW 32.02<br>SE 41.75<br>SW 63.28<br>TOTAL 45.45
**Z_axis**	6;10	
**xz_planes**	10	
**density**	0.0001	
**excluded atoms**	1,2,3,17,133,134,135,136,137,138,139,140,141,142,143,144,145,146,147,148,149,127,131,150,132,151,152,153,154,155,156,157,158,159,160	

system 1 - MCB-IN*cis*_ COOtBu-cph3
olefin (no Hs)

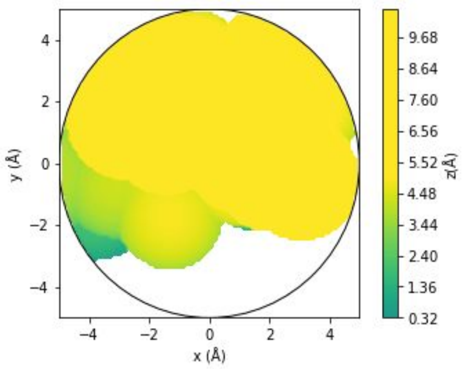

**Center:**	92	NE 30.47<br>NW42.84<br>SE 41.94<br>SW 21.78<br>TOTAL 34.26
**Z_axis**	6;10	
**xz_planes**	10	
**density**	0.0001	
**excluded atoms**	1,2,3,4, 5, 6, 7, 8, 9, 10, 11, 12, 13, 14, 15, 16, 18, 19, 20, 21, 22, 23, 24, 25, 26, 27, 28, 29, 30, 31, 32, 33, 34, 35, 36, 37, 38, 39, 40, 41, 42, 43, 44, 45, 46, 47, 48, 49, 50, 51, 52, 53, 54, 55, 56, 57, 58, 59, 60, 61, 62, 63, 64, 65, 66, 67, 68, 69, 70, 71, 72, 73, 74, 75, 76, 77, 78, 79, 80, 81, 82, 83, 84, 85, 86, 87, 88, 89, 90, 91, 92, 93, 94, 95, 96, 97, 98, 99, 100, 101, 102, 103, 104, 105, 106, 107, 108, 109, 110, 111, 112, 113, 114, 115, 116, 117, 118, 119, 120, 121, 122, 123, 124, 125	

system 1 - MCB-INtrans COOtBu-cph3
NHC (no Hs)

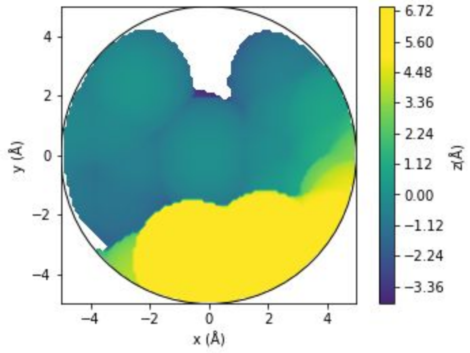

**Center:**	92	NE 38.98
**Z_axis**	6;10	NW 36.93
**xz_planes**	10	SE 41.60
**density**	0.0001	SW 67.73<br>TOTAL 46.31
**excluded atoms**	[1,2,3,17,134,135,136,137,138,139,140,141,142,143,144,145,146,147,148,149,150,127,128,131,132,133,151,152,153,154,155,156,157,158,159,160,126,129,130]	

system 1 - MCB-IN*trans*_ COOtBu-cph3
olefin (no Hs)

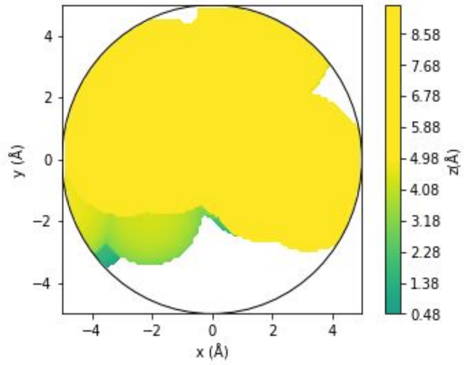

**Center:**	92	NE 24.83
**Z_axis**	6;10	NW 30.99
**xz_planes**	10	SE 39.31
**density**	0.0001	SW 21.23<br>TOTAL 29.09
**excluded atoms**	1,2,3,4, 5, 6, 7, 8, 9, 10, 11, 12, 13, 14, 15, 16, 18, 19, 20, 21, 22, 23, 24, 25, 26, 27, 28, 29, 30, 31, 32, 33, 34, 35, 36, 37, 38, 39, 40, 41, 42, 43, 44, 45, 46, 47, 48, 49, 50, 51, 52, 53, 54, 55, 56, 57, 58, 59, 60, 61, 62, 63, 64, 65, 66, 67, 68, 69, 70, 71, 72, 73, 74, 75, 76, 77, 78, 79, 80, 81, 82, 83, 84, 85, 86, 87, 88, 89, 90, 91, 92, 93, 94, 95, 96, 97, 98, 99, 100, 101, 102, 103, 104, 105, 106, 107, 108, 109, 110, 111, 112, 113, 114, 115, 116, 117, 118, 119, 120, 121, 122, 123, 124, 125	

system 1 - MCB-OUT*cis*_COOtBu-cph3
NHC (no Hs)

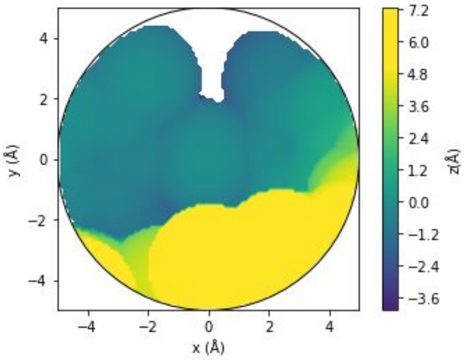

**Center:**	92	NE 40.40
**Z_axis**	6;10	NW 39.45
**xz_planes**	10	SE 45.67

**density**	0.0001	SW 64.86<br>TOTAL 47.59
**excluded atoms**	[1,2,3,17,131,132,133,134,135,136,137,138,139,140,141,142,143,144,145,146,147,127,128,149,148,150,151,152,153,155,154,156,158,157,159,160,126,129,130]	

system 1 - MCB-OUT*cis*_COOtBu-cph3
olefin (no Hs)

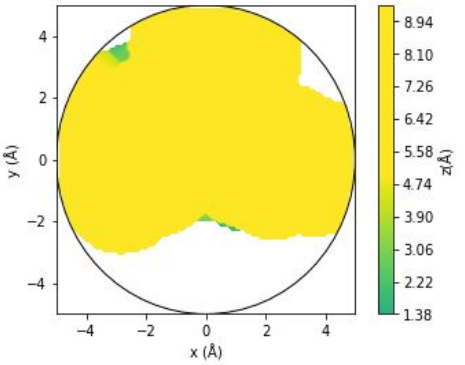

**Center:**	92	NE 22.22<br>NW 41.38<br>SE 22.72<br>SW 19.47<br>TOTAL 26.45
**Z_axis**	6;10	
**xz_planes**	10	
**density**	0.0001	
**excluded atoms**	1,2,3,4, 5, 6, 7, 8, 9, 10, 11, 12, 13, 14, 15, 16, 18, 19, 20, 21, 22, 23, 24, 25, 26, 27, 28, 29, 30, 31, 32, 33, 34, 35, 36, 37, 38, 39, 40, 41, 42, 43, 44, 45, 46, 47, 48, 49, 50, 51, 52, 53, 54, 55, 56, 57, 58, 59, 60, 61, 62, 63, 64, 65, 66, 67, 68, 69, 70, 71, 72, 73, 74, 75, 76, 77, 78, 79, 80, 81, 82, 83, 84, 85, 86, 87, 88, 89, 90, 91, 92, 93, 94, 95, 96, 97, 98, 99, 100, 101, 102, 103, 104, 105, 106, 107, 108, 109, 110, 111, 112, 113, 114, 115, 116, 117, 118, 119, 120, 121, 122, 123, 124, 125	

system 1 - MCB-OUT*trans*_COOtBu-cph3
NHC (no Hs)

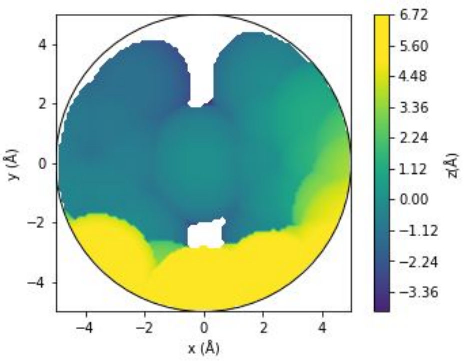

**Center:**	92	NE 46.62<br>NW 29.23<br>SE 46.63<br>SW 57.56<br>TOTAL 45.01
**Z_axis**	6;10	
**xz_planes**	10	
**density**	0.0001	
**excluded atoms**	1,2,3,17,131,132,133,134,135,136,137,138,139,140,141,142,143,144,145,146,147,127,128,149,148,150,152,151,153,155,154,156,157,158,158,160,126,129,130	

system 1 - MCB-OUT*trans*_ COOtBu-cph3
olefin (no Hs)

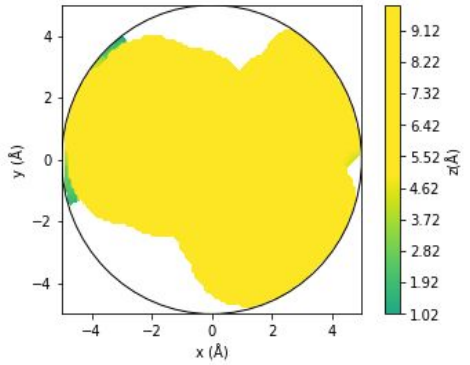

**Center:**	92	NE 27.86<br>NW 43.05<br>SE 22.25<br>SW 21.58<br>TOTAL 28.69
**Z_axis**	6;10	
**xz_planes**	10	
**density**	0.0001	
**excluded atoms**	1,2,3,4, 5, 6, 7, 8, 9, 10, 11, 12, 13, 14, 15, 16, 18, 19, 20, 21, 22, 23, 24, 25, 26, 27, 28, 29, 30, 31, 32, 33, 34, 35, 36, 37, 38, 39, 40, 41, 42, 43, 44, 45, 46, 47, 48, 49, 50, 51, 52, 53, 54, 55, 56, 57, 58, 59, 60, 61, 62, 63, 64, 65, 66, 67, 68, 69, 70, 71, 72, 73, 74, 75, 76, 77, 78, 79, 80, 81, 82, 83, 84, 85, 86, 87, 88, 89, 90, 91, 92, 93, 94, 95, 96, 97, 98, 99, 100, 101, 102, 103, 104, 105, 106, 107, 108, 109, 110, 111, 112, 113, 114, 115, 116, 117, 118, 119, 120, 121, 122, 123, 124, 125	

Table S10. Steric maps for alkyl-COOtBu (radius of 5 Å). The isocontour curves of the steric maps are in Å. The xz plane is the mean plane of the NHC ring, whereas the yz plane is the plane orthogonal to the mean plane of the NHC ring, and passing through the carbene C atom of the NHC ring. The metal is at the origin.

system 1 – MCB-IN*cis*-(alpha)alkyl-COOtBu<br>**olefin (no Hs)**		
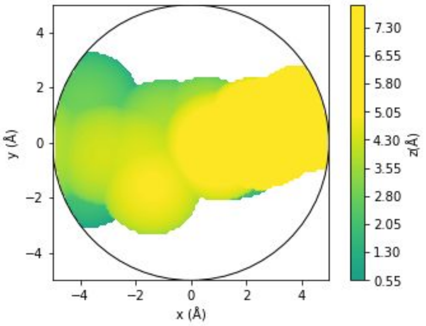		
**Center:**	92	**NE**<br>**NW**<br>**SE**<br>**SW**<br>**Total** 29.31
**Z_axis**	6;10	
**xz_planes**	10	
**density**	0.0001	
**excluded atoms**	1,2,3,4, 5, 6, 7, 8, 9, 10, 11, 12, 13, 14, 15, 16, 18, 19, 20, 21, 22, 23, 24, 25, 26, 27, 28, 29, 30, 31, 32, 33, 34, 35, 36, 37, 38, 39, 40, 41, 42, 43, 44, 45, 46, 47, 48, 49, 50, 51, 52, 53, 54, 55, 56, 57, 58, 59, 60, 61, 62, 63, 64, 65, 66, 67, 68, 69, 70, 71, 72, 73, 74, 75, 76, 77, 78, 79, 80, 81, 82, 83, 84, 85, 86, 87, 88, 89, 90, 91, 92, 93, 94, 95, 96, 97, 98, 99, 100, 101, 102, 103, 104, 105, 106, 107, 108, 109, 110, 111, 112, 113, 114, 115, 116, 117, 118, 119, 120, 121, 122, 123, 124, 125	

system 1 – MCB-IN*trans*-(alpha)alkyl-COOtBu<br>**olefin (no Hs)**		
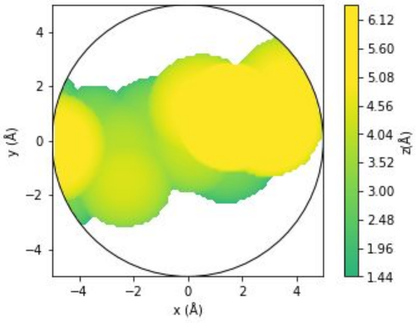		
**Center:**	92	**NE**<br>**NW**<br>**SE**<br>**SW**<br>**Total** 25.84
**Z_axis**	6;10	
**xz_planes**	10	
**density**	0.0001	
**excluded atoms**	1,2,3,4, 5, 6, 7, 8, 9, 10, 11, 12, 13, 14, 15, 16, 18, 19, 20, 21, 22, 23, 24, 25, 26, 27, 28, 29, 30, 31, 32, 33, 34, 35, 36, 37, 38, 39, 40, 41, 42, 43, 44, 45, 46, 47, 48, 49, 50, 51, 52, 53, 54, 55, 56, 57, 58, 59, 60, 61, 62, 63, 64, 65, 66, 67, 68, 69, 70, 71, 72, 73, 74, 75, 76, 77, 78, 79, 80, 81, 82, 83, 84, 85, 86, 87, 88, 89, 90, 91, 92, 93, 94, 95, 96, 97, 98, 99,	

	100, 101, 102, 103, 104, 105, 106, 107, 108, 109, 110, 111, 112, 113, 114, 115, 116, 117, 118, 119, 120, 121, 122, 123, 124, 125

system 1 – MCB-OUT*cis*-(alpha)alkyl-COOtBu
olefin (no Hs)

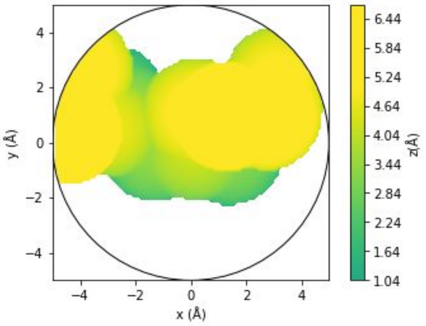

**Center:**	92	**NE**
**Z_axis**	6;10	**NW**
**xz_planes**	10	**SE**
**density**	0.0001	**SW** **Total** 25.67
**excluded atoms**	1,2,3,4, 5, 6, 7, 8, 9, 10, 11, 12, 13, 14, 15, 16, 18, 19, 20, 21, 22, 23, 24, 25, 26, 27, 28, 29, 30, 31, 32, 33, 34, 35, 36, 37, 38, 39, 40, 41, 42, 43, 44, 45, 46, 47, 48, 49, 50, 51, 52, 53, 54, 55, 56, 57, 58, 59, 60, 61, 62, 63, 64, 65, 66, 67, 68, 69, 70, 71, 72, 73, 74, 75, 76, 77, 78, 79, 80, 81, 82, 83, 84, 85, 86, 87, 88, 89, 90, 91, 92, 93, 94, 95, 96, 97, 98, 99, 100, 101, 102, 103, 104, 105, 106, 107, 108, 109, 110, 111, 112, 113, 114, 115, 116, 117, 118, 119, 120, 121, 122, 123, 124, 125	

system 1 – MCB-OUT*trans*-(alpha)alkyl-COOtBu
olefin (no Hs)

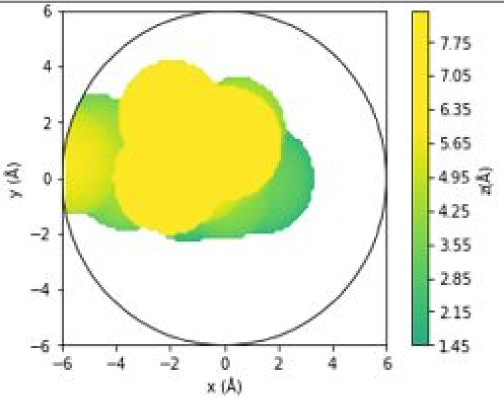

**Center:**	92	**NE**
**Z_axis**	6;10	**NW**
**xz_planes**	10	**SE**
**density**	0.0001	**SW** **Total** 24.62
**excluded atoms**	1,2,3,4, 5, 6, 7, 8, 9, 10, 11, 12, 13, 14, 15, 16, 18, 19, 20, 21, 22, 23, 24, 25, 26, 27, 28, 29, 30, 31, 32, 33, 34, 35, 36, 37, 38, 39, 40, 41, 42, 43, 44, 45, 46, 47, 48, 49, 50, 51, 52, 53, 54, 55, 56, 57, 58, 59, 60, 61, 62, 63, 64, 65, 66, 67, 68, 69, 70, 71, 72, 73, 74, 75, 76, 77, 78, 79, 80, 81, 82, 83, 84, 85, 86, 87, 88, 89, 90, 91, 92, 93, 94, 95, 96, 97, 98, 99, 100, 101, 102, 103, 104, 105, 106, 107, 108, 109, 110, 111, 112, 113, 114, 115, 116, 117, 118, 119, 120, 121, 122, 123, 124, 125	

Table S11. Correlation between $\%V_{Bur}$ and Energies (relative Gibbs energies in kcal/mol).

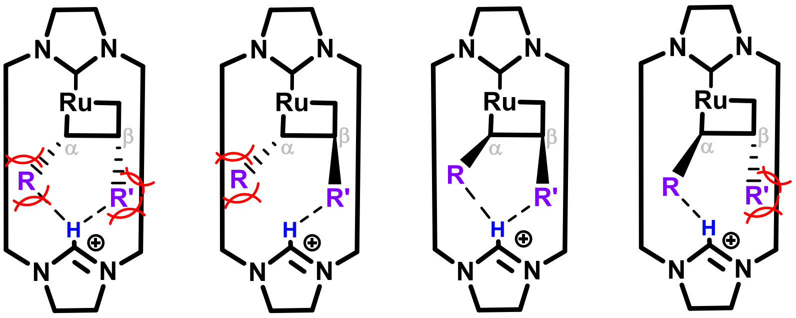

		IN-*cis*	IN-*trans*	OUT-*cis*	OUT-*trans*
**-CPh$_3$**	**(R')**	19.5	4.1	14.6	16.9
**-(CH$_2$)$_5$CH$_3$**	**(R')**	16.3	-2.4	9.4	2.8
**-(CH$_2$)$_5$CH$_3$**	**(R)**	0.9	2.9	2.4	2.5
**$\%V_{Bur}^{-CPh3(R')}$**		34.26	29.09	26.45	28.69
**$\%V_{Bur}^{-(CH2)5CH3(R')}$**		29.31	25.84	25.67	24.62
**$\%V_{Bur}^{-(CH2)5CH3(R)}$**		22.93	26.00	22.82	22.89

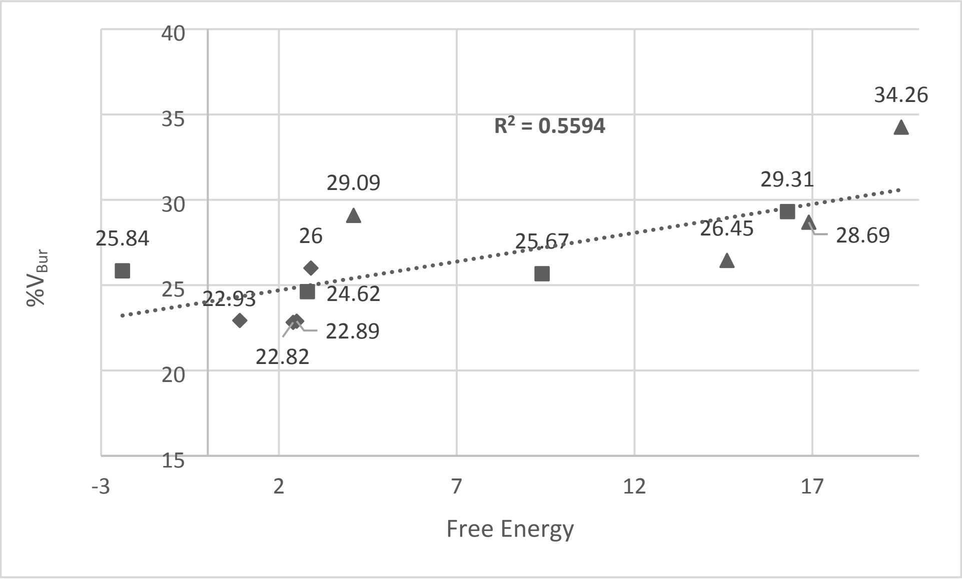

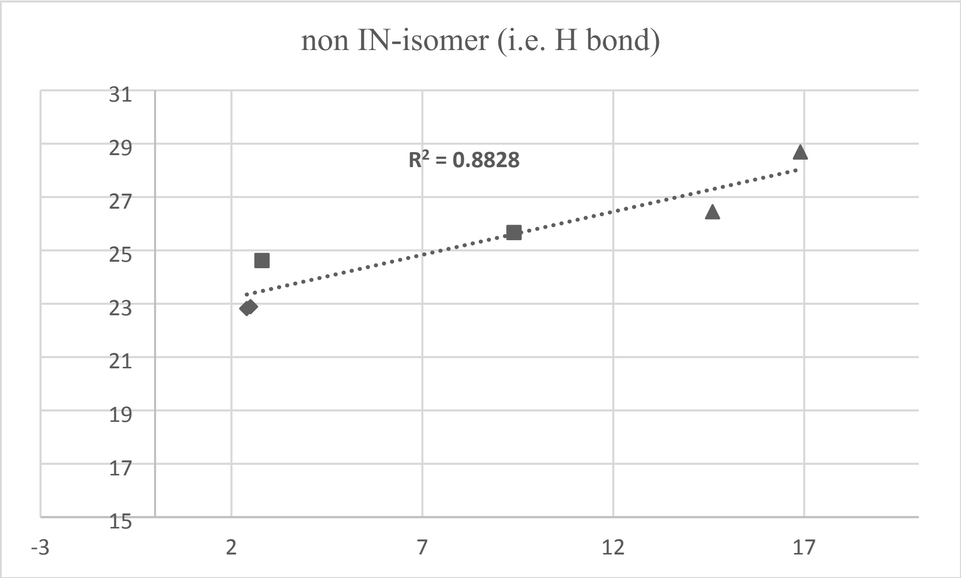
non IN-isomer (i.e. H bond)
R² = 0.8828
31
29
27
25
23
21
19
17
15
-3
2
7
12
17

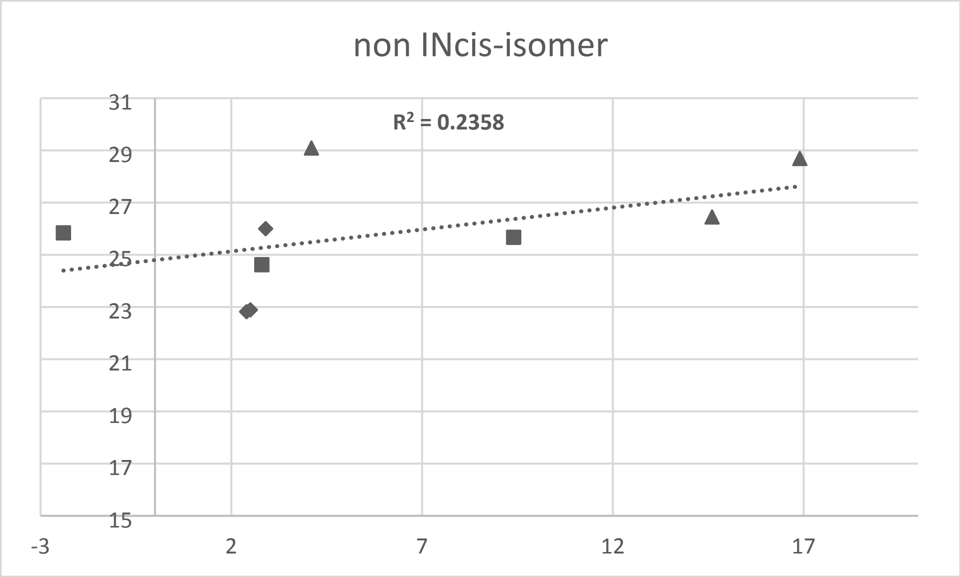
non INcis-isomer
R² = 0.2358
31
29
27
25
23
21
19
17
15
-3
2
7
12
17

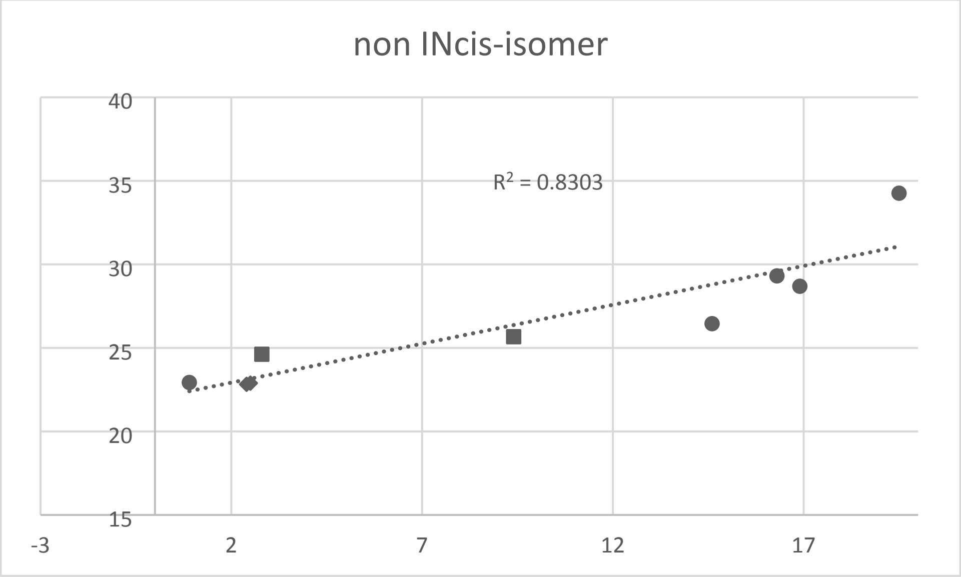
non INcis-isomer
R² = 0.8303
40
35
30
25
20
15
-3
2
7
12
17

Figure S1. 2D NCI Plots, and correlations.

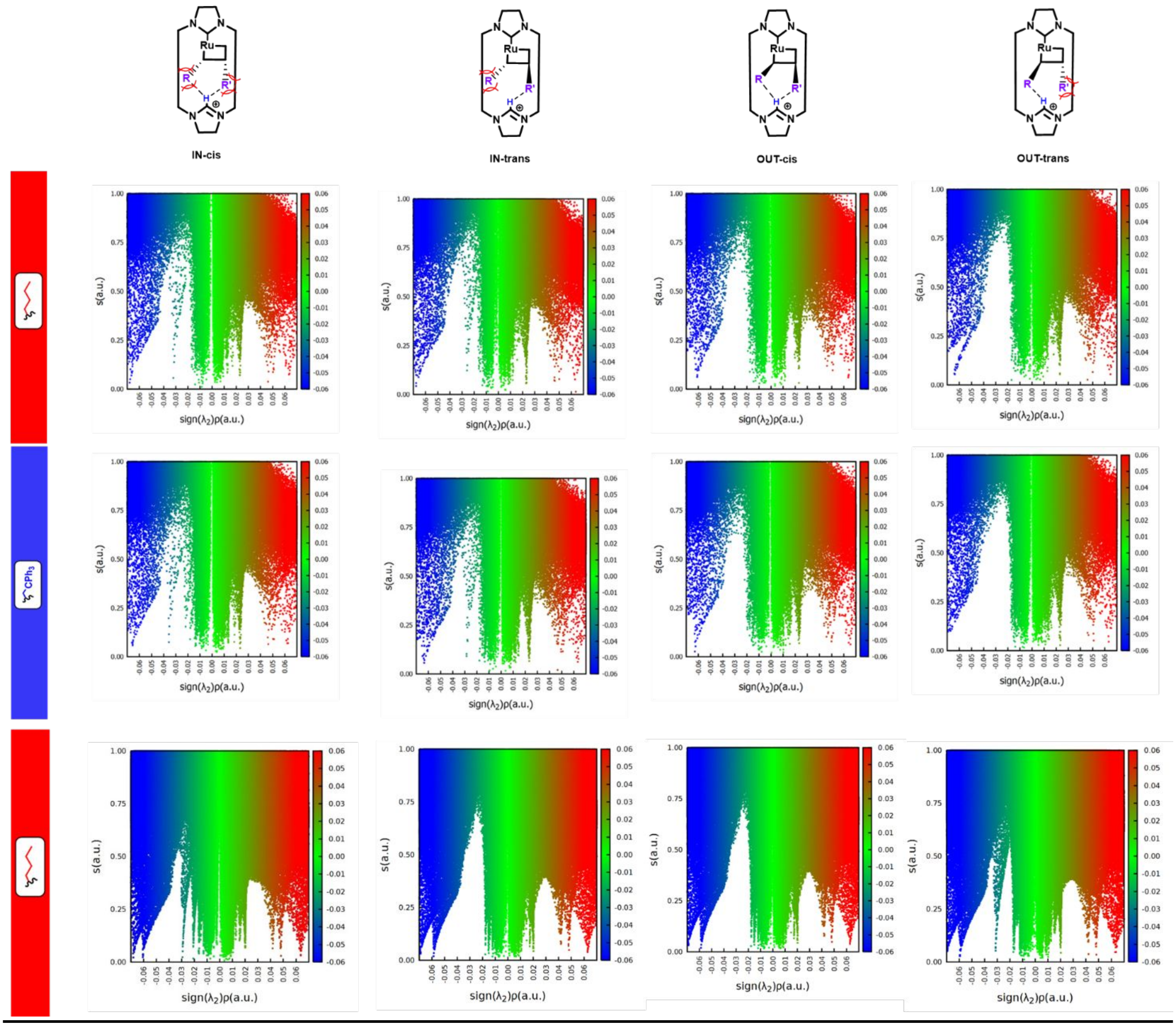

Figure S2. Correlations between relative energies in kcal/mol and %V_{Bur}.

	E_{gas}	G_{gas}	E_{solv}	G_{solv}	-NHC-	-olefin-
**IN-*cis***	-44.07	-15.46	-13.98	14.63	44.83	29.31
**In-*trans***	-56.80	-31.20	-29.80	-4.10	46.76	5.84
**OUT-*cis***	-42.11	-16.45	-17.96	7.69	49.92	25.67
**OUT-*trans***	-40.42	-15.58	-23.73	1.11	46.20	24.62

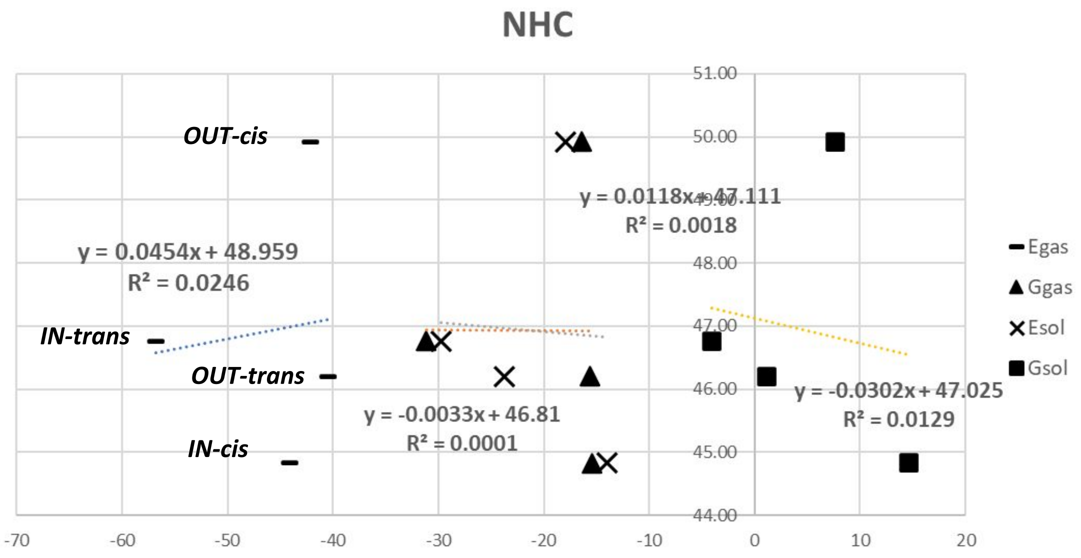

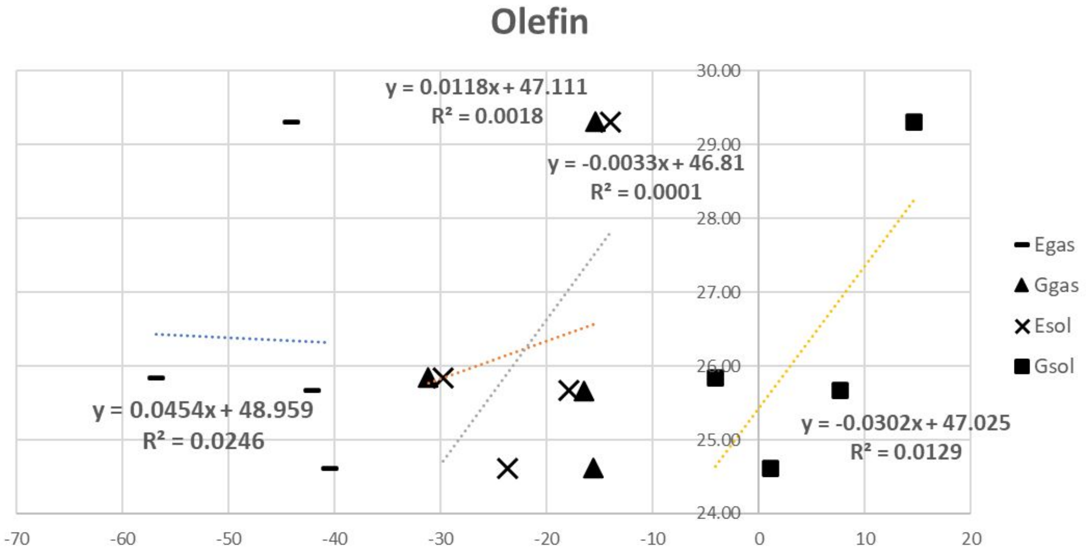

	E_{gas}	G_{gas}	E_{solv}	G_{solv}	-NHC-	-olefin-
**IN-*cis***	-57.06	-25.44	-13.76	1786	45.45	34.26
**In-*trans***	-61.77	-33.06	-26.28	2.43	46.31	29.09
**OUT-*cis***	-47.96	-19.99	-15.07	12.90	47.95	26.45
**OUT-*trans***	-46.56	-17.99	-13.38	15.18	45.01	28.69

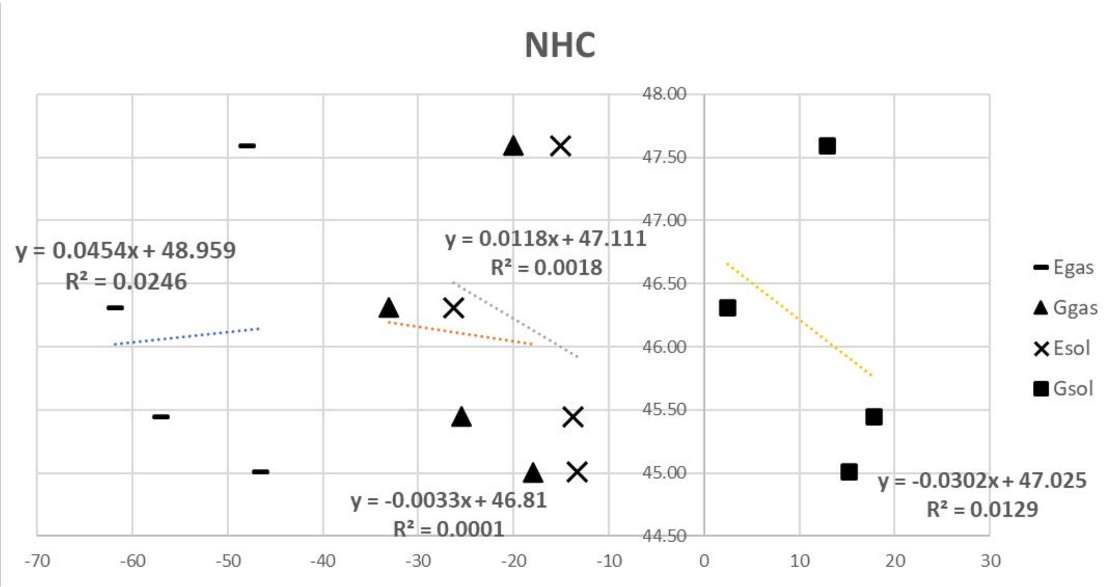

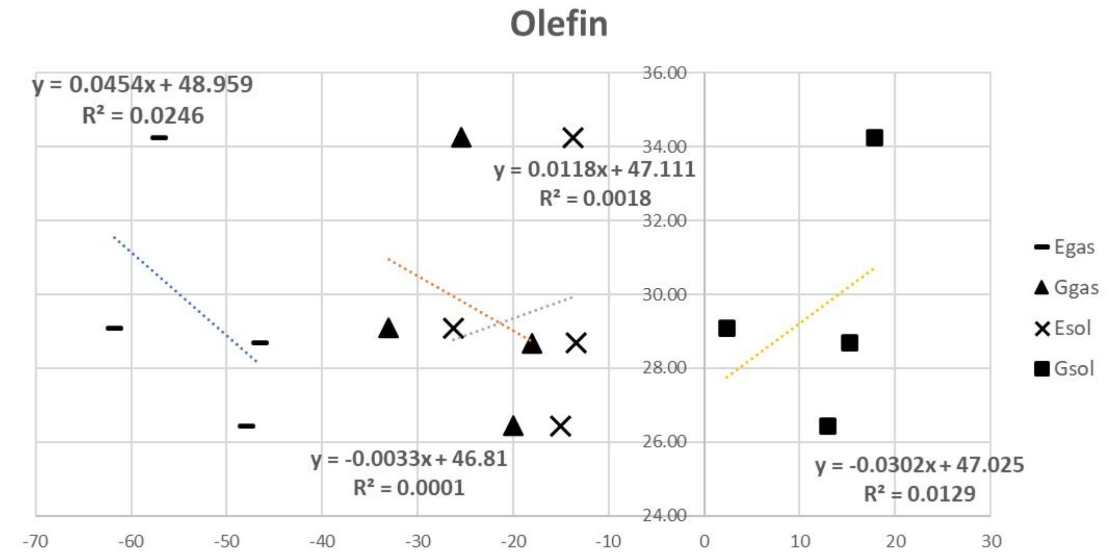

Statistical tests (Simplified):

E_{gas}	Determination coefficient R^2	Typical error	*m*	n	Lim. Inf	Lim. Sup.
NE	0.07	8.73	-0.37	-36.05	-17.91	17.18
NW	1.00	0.11	0.62	-65.55	0.52	0.71
SE	1.00	0.42	-0.52	-32.52	-0.83	-0.21
SW	0.19	8.14	-1.23	-18.68	-33.60	31.14

E_{solv}	Determination coefficient R^2	Typical error	*m*	n	Lim. Inf	Lim. Sup.
NE	0.22	4.36	0.51	-31.04	-2.42	3.45
NW	0.10	4.69	-0.15	-13.92	-1.52	1.23
SE	0.45	3.66	0.21	-24.58	-0.49	0.90
SW	0.13	4.62	-0.77	-2.25	-6.99	5.44

G_{gas}	Determination coefficient R^2	Typical error	*m*	n	Lim. Inf	Lim. Sup.
NE	0.05	6.72	-0.23	-13.15	-13.75	13.28
NW	1.00	0.38	0.47	-34.51	0.14	0.80
SE	1.00	0.03	-0.40	-9.36	-0.42	-0.38
SW	0.22	6.07	-1.02	2.95	-25.16	23.12

E_{solv}	Determination coefficient R^2	Typical error	*m*	n	Lim. Inf	Lim. Sup.
**NE**	0.16	6.22	0.59	-6.16	-3.60	4.77
**NW**	0.22	5.96	-0.31	18.10	-2.05	1.44
**SE**	0.65	4.00	0.34	-1.59	-0.42	1.10
**SW**	0.03	6.67	-0.52	19.11	-9.49	8.44

ALPHA:

	E_{gas}	G_{gas}	E_{solv}	G_{solv}	-NHC-	-olefin-
**IN-*cis***	-50.17	-24.53	-26.44	-0.80	46.70	17.48
**In-*trans***	-50.29	-24.90	-24.18	1.21	49.41	20.58
**OUT-*cis***	-43.23	-18.44	-24.04	0.75	49.20	17.33
**OUT-*trans***	-50.22	-25.34	-24.04	0.84	46.70	17.54

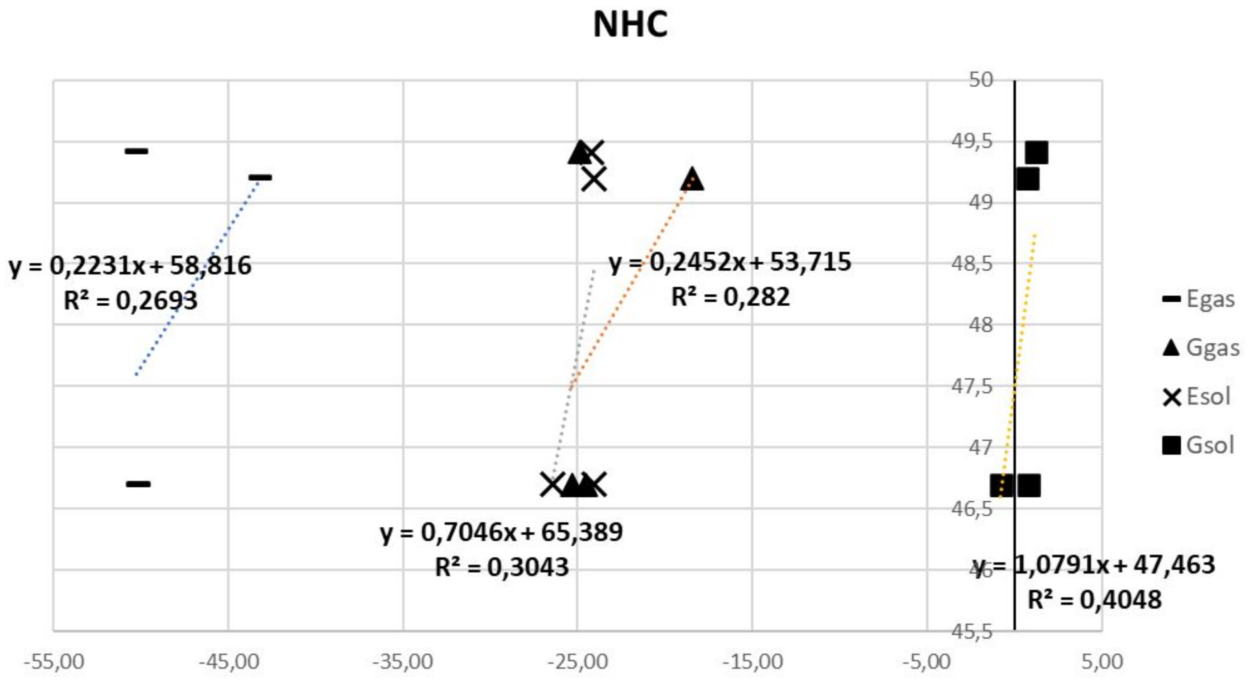

Quadrants:

R^2	NE	NW	SE	SW	Total
DeltaG	0.70	0.60	0.37	0.09	0.41

Statistical treatment of the higher correlated values (NE).

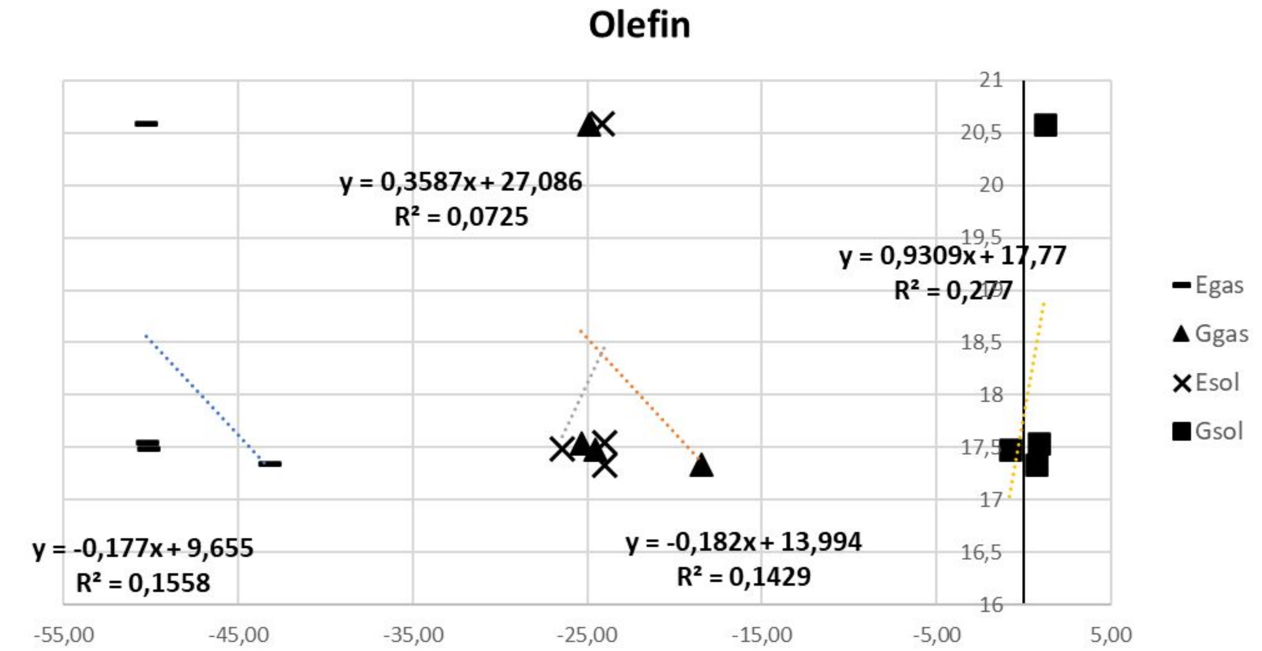

Quadrants:

R^2	NE	NW	SE	SW	Total
DeltaG	0.58	0.03	0.95	0.01	0.29

Statistical treatment of the higher correlated values (SE).

Considering that the *y-axis* corresponds to the %V_{Bur}. and the *x-axis* corresponds to the Energy, a negative slope (*Coefficient: Variable X* = -0.44310) does not fit with the experimental data. Increasing the energy cannot come with a decrease of the %V_{Bur} value (i.e. sterics).

Using all the %V_{Bur} values in Figure S3 we do not fulfill a clear agreement either.

Figure S3. Correlations between relative energies in kcal/mol and %V_{Bur}.

	~olefin~	E_{gas}	G_{gas}	E_{sol}	G_{sol}	V_{TOT}
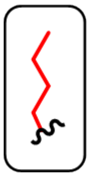	**IN-*cis***	-44.07	-15.46	-13.98	14.63	29.31
	**IN-*trans***	-56.8	-31.2	-29.8	-4.1	25.84
	**OUT-*cis***	-42.11	-16.45	-17.96	7.69	25.67
	**OUT-*trans***	-40.42	-15.58	-23.73	1.11	24.62

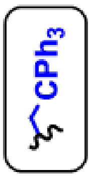	**IN-*cis***	-57.06	-25.44	-13.76	17.86	34.26
	**IN-*trans***	-61.77	-33.06	-26.28	2.43	29.09
	**OUT-*cis***	-47.96	-19.99	-15.07	12.9	26.45
	**OUT-*trans***	-46.54	-17.99	-13.38	15.18	28.69
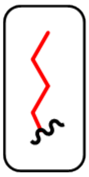	**IN-*cis***	-50.17	-24.53	-26.44	-0.8	17.48
	**IN-*trans***	-50.29	-24.9	-24.18	1.21	20.58
	**OUT-*cis***	-43.23	-18.44	-24.04	0.75	20.58
(alpha)	**OUT-*trans***	-50.22	-25.34	-24.04	0.84	17.54

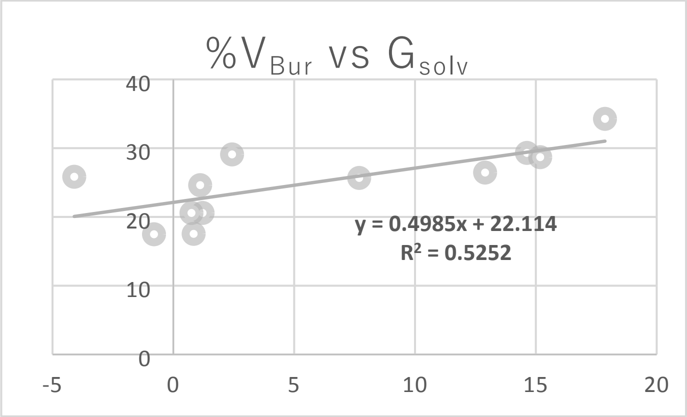

Supplement: Supplementary file 1 [file ic5c03590_si_001.pdf]
